# Biofilm-associated genes in *Staphylococcus aureus*: role in chronic infections and therapeutic challenges

**DOI:** 10.3389/fmicb.2026.1915213

**Published:** 2026-10-05

**Authors:** Mohammad Asim Azhar, Tanveer Ahmad Mir, Sana Ahmed, Abdullah M. Assiri, Dieter C. Broering

**Affiliations:** Organ Transplant Center of Excellence, King Faisal Specialist & Research Center, Riyadh, Saudi Arabia

**Keywords:** antibiotic tolerance, antibiofilm therapies, biofilm-associated genes, chronic infections, *Staphylococcus aureus*

## Abstract

One of the major contributors to persistent healthcare and community-associated infections is *S. aureus*, which has a remarkable ability to form biofilms that protect it against the host immune system and antimicrobial therapy. In recent years, it has become clear that biofilm development is a highly orchestrated and dynamic process, controlled by complex genetic networks, transcriptional regulators, two-component systems (TCSs), and environmental signals. The present review covers the molecular mechanisms involved in the initiation, maturation, maintenance, and dispersal of biofilms in general, and in particular, the coordinated functions of adhesins, the *icaADBC* operon, components of the extracellular matrix (ECM), global regulators (*agr*, *sarA*, and *sigB*), and two-component regulatory systems, as well as emerging insights from transcriptomic studies on biofilm adaptation. We then review the roles of these regulatory pathways in metabolic heterogeneity, antibiotic tolerance, immune evasion, and persistence in clinically relevant biofilm-associated infections, including infective endocarditis, osteomyelitis, prosthetic joint infection, catheter-associated infection, and diabetic foot infection. The review further outlines the latest developments in antibiofilm therapeutics, including matrix-degrading enzymes, quorum-sensing inhibitors, antimicrobial peptides, bacteriophage-based therapeutics, lysins, nitric oxide donors, nanoparticle-assisted therapeutics, and CRISPR-Cas-based antimicrobials. Incorporating molecular regulation into infection-specific pathogenesis and emerging therapeutic strategies, this review offers a new perspective on the biology of *S. aureus* biofilms and highlights opportunities to develop mechanism-based interventions to combat chronic biofilm-associated infections.

## 1 Introduction

*Staphylococcus aureus (S. aureus)* is an opportunistic Gram-positive pathogen which causes a wide range of community- and healthcare-associated infections, including superficial skin and soft tissue infections, life-threatening infections such as bacteremia, infective endocarditis, osteomyelitis, pneumonia, and device-associated infections (Kobayashi et al., 2024; McGuire et al., 2024; Saleem et al., 2026). The extensive virulence attributes, astounding adaptability to various host environments, and increasing antimicrobial resistance of *S. aureus* are believed to be responsible for the remarkable clinical success (Ahator et al., 2024). Importantly, its capacity to form biofilms on host tissues and implanted medical devices is a mechanism of persistent infection, provides protection from host immune responses and antimicrobial therapy, and plays a major role in chronic and recurrent disease (Van Roy et al., 2024).

Biofilm formation is a highly orchestrated process, not just by individual virulence factors, but by complex networks. The temporal control of adhesion molecules, extracellular matrix (ECM) production, quorum-sensing, stress-response pathways, and metabolic adaptation to constantly changing environmental conditions are all crucial for the successful establishment and persistence of biofilms (Costa et al., 2024; Syed et al., 2024). While many genes and regulatory systems have been identified as contributing to biofilm formation and implicated in various clinical infections, their interactions and communication across different stages of biofilm development and during these infections remain poorly understood. This complexity has made it very difficult to identify effective antibiotics that can treat biofilms.

Mature biofilm formation in *S. aureus* follows distinct phases, i.e., initial attachment, accumulation/maturation, and detachment/dispersal. The surface adhesins comprise microbial surface components recognizing adhesive matrix molecules (MSCRAMMs), especially fibronectin-binding proteins (FnbA/B), clumping factors (ClfA/B), and the collagen-binding adhesin (*Cna*), which bind to host tissues and artificial surfaces (Moormeier and Bayles, 2017). The accumulation stage is defined by synthesizing extracellular polysaccharides (caused mainly by the *icaADBC* operon, whose genes are needed in poly-N-acetylglucosamine (PNAG) production (Schwartbeck et al., 2024).

The last step, biofilm dispersal, leads to bacteria leaving the biofilm and invading new areas, helping spread the infection. Dispersal can be induced by nutrient starvation, oxidative stress, or signaling through the accumulation of autoinducing peptides (AIPs), and is catalyzed by bacterial proteases (SspA, Aur), nucleases (Nuc), and surfactant-like molecules, e.g., phenol-soluble modulins (PSMs; Kaplan, 2010). The *agr* system has been identified as a molecular regulator of biofilm formation and dispersal by silencing adhesins and enhancing dispersal factor expression (Arciola et al., 2015). The molecular structure of each biofilm phase is a crucial factor in developing stage-specific therapeutic interventions, particularly for drug-resistant and relapsing *S. aureus* infections (Tuon et al., 2023).

The ability of *S. aureus* to form biofilms is a key factor in the development and recurrence of infections, especially in cases involving indwelling medical devices and compromised host tissues (Beenken and Smeltzer, 2025). This stubbornness is a major cause behind the treatment failure observed in prosthetic joint infections, endocarditis, diabetic foot ulcers, and chronic wound infections. In the presence of biofilm, the bacteria are resistant to antibiotics that may be up to 1,000 times more resistant than when they are in the planktonic form, which is mainly attributed to limited drug penetration, modified microenvironments (e.g., low pH and oxygen), activation of stress response mechanisms, and efflux pumps (Almatroudi, 2025).

Besides, biofilms act as a vehicle of bacterial spread and relapse. Consistent findings from clinical studies indicate that the whole genomes of biofilm *S. aureus* strains are associated with prolonged hospital stays, patient re-admission after surgery, and increased morbidity (Rong et al., 2025). In device-associated infections, it is nearly always impossible to resolve the problem with standard antimicrobial therapy alone, without partial or total device removal, highlighting the shortcomings of standard drug therapy (Mishra et al., 2024). Biofilm also facilitates the horizontal transfer of nucleic acids, including resistance and virulence genes, thereby further increasing *S. aureus*’s adaptive capacity. Notably, cells embedded in biofilms may periodically release planktonic cells via controlled dispersal mechanisms, thereby creating new foci of infection and sustaining the infectious cycle (Shree et al., 2023).

Research in genomics, transcriptomics, and molecular microbiology has greatly improved our understanding of the regulatory architecture that drives *S. aureus* biofilm development over the past few years. These findings, however, are scattered across the literature on single regulators, environmental cues, clinical phenotypes, and therapeutic approaches. Therefore, a unified molecular and temporal understanding of biofilm persistence that combines transcriptional adaptation, disease-specific biofilm biology, and translational antibiofilm approaches is still required to offer a comprehensive understanding of persister biofilms and therapeutic targets for future research.

Individual components of *S. aureus* biofilm biology, such as the molecular mechanisms of biofilm formation, regulatory pathways that control biofilm stability, transcriptomic changes, and novel therapeutic approaches, have been the subject of several comprehensive reviews. But these studies have largely been directed at specific biological or therapeutic aspects in isolation. This review takes an integrative approach to link molecular regulation with environmental adaptation, temporal reprogramming of transcription, clinically relevant biofilm phenotypes, and new antibiotic strategies for targeting biofilms in a single framework. Special focus is given to the connectivity among regulatory networks at each stage, as well as to chronic infection, antimicrobial tolerance, and therapeutic translation.

## 2 Molecular mechanisms and key genes involved in biofilm formation

### 2.1 Adhesion and attachment phase: role of icaADBC, fnbA, fnbB, clfA, clfB, cna

The first critical point of *S. aureus* biofilm is the adhesion and attachment to the biotic or abiotic surface. The significant determinants of this phase are surface-expressed proteins, such as MSCRAMMs, which enable *S. aureus* to bind to host ECM proteins, including fibronectin, fibrinogen, and collagen (Heilmann, 2011). These binding processes allow the bacteria to colonize tissues and long-term implanted medical devices and to initiate a transition from planktonic to sessile communities. The most critical molecules of this type are the FnbA/B, which mediate interactions with fibronectin and endothelial cells and are also very important for the colonization of infective endocarditis and osteomyelitis. The ClfA/B bind fibrinogen and facilitate early bacterial cell aggregation (Vercellotti et al., 1985). Also, the *icaADBC* operon encodes enzymes that synthesize PNAG, a polysaccharide that serves as an intercellular adhesin (PIA) important for irreversible attachment to surfaces and cell–cell adhesion. *icaADBC*–PNAG/PIA is an important pathway for matrix production; however, it is not always required for *S. aureus* biofilm formation. Many clinical MRSA and MSSA isolates are highly matrix-heterogeneous, with strains producing biofilms containing both proteinaceous and extracellular DNA (eDNA) components that serve as important structural components without PNAG/PIA. Proteins associated with the cell surface, including SasG, FnbA/B, protein A, and, in bap-positive strains, biofilm-associated protein (Bap), may facilitate intercellular accumulation and matrix stabilization in these phenotypes independently of ica. eDNA can also serve as an additional structural framework and link to surface-bound proteins, reinforcing the biofilm’s structure. Importantly, the relative contribution of PNAG, protein, and eDNA is strain-, genotype-, and environment-dependent and not fixed, and therefore, *S. aureus* biofilms must be considered compositionally heterogeneous and not exclusively PNAG-dependent (Fitzpatrick et al., 2005; Merino et al., 2009; O’Neill et al., 2008).

While individual adhesins have traditionally been studied in isolation, recent evidence suggests that biofilm establishment is a coordinated, partially overlapping activity of adhesins and does not depend on a single cell-surface protein (Ahator et al., 2024). The members of the MSCRAMM family, including ClfA, ClfB, FnBPA/B, Cna, and SasG, bind to various host ECM ligands, allowing *S. aureus* to colonize a broad spectrum of host tissues and implanted biomaterials (Bhattacharya et al., 2026). The functional redundancy of these adhesins means that if one is not expressed or is expressed at a reduced level, another can substitute for it in many cases, making the bacteria more adaptable across different environments. Furthermore, the role of each adhesin is different depending on the infection niche. FnBPs and ClfA are then more important for the binding to fibrinogen- and fibronectin-coated surfaces, and are thus involved in a predominant way during infective endocarditis and device-associated infections, while Cna is more important for binding to collagen-rich tissues, such as bone, during osteomyelitis (Madani et al., 2017; Foster, 2019). The synthesis of PNAG/PIA and eDNA, and of matrix-associated proteins that promote intercellular cohesion and biofilm stability, is complementary to adhesin-mediated attachment as biofilm development continues and, together, contributes to biofilm stability. The expression is regulated globally by sarA, sigB, agr, and several two-component systems (TCSs), and is directly affected by environmental conditions (shear stress, nutrients, iron, host plasma proteins, and abiotic surface properties; Crosby et al., 2024; Syed et al., 2024). Hence, the ability of a biofilm to form depends on integrating multiple pathways and adhesins rather than on any single biofilm-associated gene.

Table 1 identifies essential genes and their protein products involved in the adhesion and attachment phase of *S. aureus* biofilm formation, a crucial step in the pathogenesis of chronic and device-related infections. The adhesins (including FnbA/B, ClfA/B, and cna) bind to host ECM proteins (fibronectin, fibrinogen, and collagen) and are associated with colonization of tissues and medical devices (Madani et al., 2017). Spa and Sdr family proteins evade the immune system and bind to epithelial cells, thereby increasing their survival in hostile environments, such as the bloodstream and the nasal mucosa (Idrees et al., 2021).

**Table 1:** Key genes involved in adhesion and attachment phase of *S. aureus* biofilm formation.

Gene/operon	Protein product	Target ligand/function	Microbiological and clinical relevance	References
*fnbA, fnbB*	FnbA/B	Bind fibronectin; mediate adherence to endothelial and epithelial cells	Essential in infective endocarditis, osteomyelitis, and internal abscesses	Speziale and Pietrocola (2020)
*clfA, clfB*	Clumping factors A, B	Bind fibrinogen; promote aggregation and immune evasion	Involved in catheter-related bloodstream infections and clot colonization	Liesenborghs et al. (2018)
*cna*	Collagen-binding adhesin	Binds to type I, II, and IV collagen in host tissues	Critical in orthopedic implants and bone-associated infections	Montanaro et al. (1999)
*spa*	Staphylococcal protein A	Binds Fc region of IgG and von Willebrand factor	Aids immune evasion and contributes to adherence under shear stress	O’Seaghdha et al. (2006)
*sdrC, sdrD, sdrE*	Serine-aspartate repeat proteins	Bind to desquamated epithelial cells and matrix proteins	Important in nasal colonization and skin adhesion	Askarian et al. (2016)
*ebpS*	Elastin-binding protein	Binds elastin in connective tissues	Facilitates colonization of lung and vascular tissues	Halsey et al. (2023)
*sasG*	Surface-associated protein G	Promotes intercellular adhesion, particularly in *ica*-independent biofilms	Promotes intercellular accumulation in PNAG/PIA-independent, proteinaceous biofilms; associated with persistent colonization and biofilm maturation	Peng et al. (2022)
*eDNA*	Extracellular DNA	Provides structural scaffold and promotes intercellular cohesion	Contributes to PNAG-independent and mixed-matrix biofilms; supports biofilm architecture & persistence	Mann et al. (2009); Bowden et al. (2024)
*icaADBC*	PNAG synthase (PIA biosynthesis)	Synthesizes PNAG for cell–cell adhesion	Central to irreversible attachment and accumulation of mature biofilm matrix	Nguyen et al. (2020)
*atl*	Autolysin	Facilitates initial adhesion via surface hydrophobic interactions	Aids attachment to abiotic surfaces and modulates cell lysis during biofilm growth	Sharma et al. (2023)

SasG is especially crucial for PNAG-independent biofilm development, as proteolytic cleavage exposes adhesive regions that enable homophilic interactions between adjacent cells via zinc-dependent mechanisms, without the need for PNAG/PIA (Yonemoto et al., 2019). Similarly, FnbA/B can facilitate the production of proteinaceous biofilm under certain conditions. Protein A has also been experimentally demonstrated to promote an ica-independent biofilm growth phenotype, while Bap can induce a strong PNAG-independent biofilm growth in the Bap-positive *S. aureus* lineages (Rudolph et al., 2026). The eDNA is another non-polysaccharide matrix scaffold that contributes to the matrix’s spatial structure and cohesion. In this way, protein- and eDNA-dependent mechanisms can counteract a decrease or complete absence of PNAG production, creating biofilms with a different matrix composition and potentially differing in their sensitivity to matrix-targeted therapeutic methods (Rana et al., 2026).

Production of PNAG, one of the major polysaccharides on the biofilm matrix that holds together bacterial cells, is similarly essential to the presence of the *icaADBC* operon. Also, an autolysin (*atl)* contributes to biofilms’ early attachment and structural integrity (Table 1). The cumulative gene set highlights the multifactorial and tightly controlled nature of *S. aureus* attachment, setting the stage for biofilm formation, survival, and antimicrobial resistance (Aboelnaga et al., 2024).

Multiple global regulatory circuits control the expression of adhesins involved in initial attachment, enabling rapid adaptation to varying environmental conditions. Of these, SarA, a DNA-binding protein, serves as a positive central regulator, controlling the expression of multiple surface adhesins such as ClfA, FnBPA/B and SasG, and simultaneously repressing the expression of extracellular proteases that could otherwise degrade these proteins (Xiong et al., 2004; Beenken et al., 2025). In addition, under nutrient limitation, osmotic stress, and other environmental stresses, the alternative sigma factor (SigB) further promotes early biofilm formation by promoting stress adaptation and boosting the expression of adhesins and the *icaADBC* operon (Rachid et al., 2000). At low cell density, however, activation of the *agr*-quorum-sensing system is relatively low, allowing stable surface attachment; when the cell density increases, the expression of several adhesins is repressed by activation of RNAIII via agrA, whereas the expression of PSMs, proteases, and nuclease is induced, which is associated with biofilm remodeling and dispersal (Queck et al., 2008). Early attachment is also a result of TCSs. SaeRS regulates the expression of multiple surface proteins associated with virulence, and ArlRS regulates autolysis, surface protein expression, and eDNA production, which are necessary to establish a stable biofilm in the presence of host signals. Furthermore, the *icaADBC* operon is negatively regulated by the transcriptional repressor *IcaR*, so it is not activated until environmental signals are right for irreversible biofilm formation (Schwartbeck et al., 2024; Zhou et al., 2024). Together, these regulatory systems coordinate environmental signals and adhesin expression, allowing for the proper first phase of adhesion to be tightly regulated for the second phase of mature biofilm.

### 2.2 Biofilm maturation: role of sarA, agr, sigB, and icaR

The second stage of the *S. aureus* biofilm is the maturation phase, characterized by elevated biomass, structural complexity, a three-dimensional architecture, and a strengthened ECM, which facilitates antimicrobial tolerance and immune evasion (Schilcher and Horswill, 2020). Global transcriptional regulators (*sarA*, *agr*, *sigB*, and *icaR*) tightly control this stage, coordinating genes that mediate intercellular adhesion, matrix production, and virulence (Aboelnaga et al., 2024). The *sarA* gene is a key positive regulator of biofilm accumulation and biofilm matrix stability, but it has additional effects beyond activating the *icaADBC* operon. *sarA* is a global regulator that inhibits extracellular proteases and stimulates the production and stability of several adhesion factors and matrix proteins, thereby favoring both PNAG-dependent and PNAG-independent biofilm phenotypes. Therefore, the increased production of PIA/PNAG cannot be attributed solely to *sarA*’s role in biofilm formation (Beenken et al., 2025). In contrast, the *agr* quorum-sensing system, which negatively regulates biofilm maturation, promotes biofilm dispersion and the production of surfactant-like compounds such as PSMs, thereby inhibiting cell–cell interactions. *sigB*, a second factor, enhances biofilm resilience under stress by activating genes involved in autolysis and cell wall remodeling, thereby reinforcing matrix architecture (Cue et al., 2013). The IcaR protein binds to the *icaADBC* operon promoter and negatively regulates biosynthesis and accumulation of PIA. The interaction of these regulators enables the formation of a clear biofilm structure that is both dynamic and sufficiently stable to withstand host defenses and antibiotics when infections become chronic and relapsing (Hoang et al., 2019).

Global regulators do not act alone; they work together in a regulatory network to coordinate biofilm development in response to bacterial population density and environmental conditions. sarA directly binds to the promoters of several biofilm-associated genes, including the *icaADBC* operon, and can also directly repress genes encoding extracellular proteases that help maintain matrix integrity (Shaw et al., 2004; Cerca et al., 2008). The activation of the *agr*-system, on the other hand, leads to the phosphorylation of the response regulator agrA and binding of the P2 and P3 promoters. The P2 promoter positively feeds back to amplify the expression of the *agr* operon, while transcription from the P3 promoter produces RNAIII, the major *agr* effector molecule (Novick et al., 1993). Unlike transcription factors, RNAIII modulates gene expression post-transcriptionally by base-pairing with its target mRNAs, thereby affecting ribosome accessibility to the mRNA or its stability (Morfeldt et al., 1995). This mechanism downregulates the expression of several surface adhesins and indirectly upregulates the expression of secreted toxins and factors involved in dissemination and dispersal, moving the organisms from a sessile “biofilm” stage to a planktonic stage of dissemination (Vuong et al., 2000). Thus, biofilm maturation relies not only on sarA but also on RNAIII (which activates dispersal programs), with a dynamic interplay between the two rather than on either alone (Mrak et al., 2012).

This stage-wise regulation model is also supported by evidence from clinical isolates. Chronic osteomyelitis, prosthetic joint infections, and device-associated infections are often associated with persisting isolates that may exhibit lower agr activity or natural mutations in the *agr* locus that leads to increased expression of adhesion factors, increased ECM production, and greater biofilm stability (Tuchscherr et al., 2020). Alternatively, isolates exhibiting either complete or robust agr signaling tend to have higher expression of secreted toxins and dispersal factors, which are associated with invasive disease and dissemination (Yamazaki et al., 2024b). However, there is significant strain-to-strain variability among clinical isolates, which may be attributed to differences in the clonal lineage, niche of infection, environmental conditions, and to other regulatory networks that further influence the biofilm persister/disperser balance, including sigB, saeRS, ArlRS, and GraRS (Cassat et al., 2006).

An overview of the major molecular regulators that control the maturation step of biofilm development in *S. aureus,* with emphasis on the genetic targets, regulatory properties, and their impact on biofilm stability, is presented in Table 2. The *sarA* gene encodes a global transcriptional activator that promotes biofilm maturation by upregulating the *icaADBC* operon, which encodes the PIA cargo, and downregulating extracellular proteases that may otherwise degrade the matrix (Zielinska et al., 2012). Conversely, the *agr* system, a quorum-sensing regulator that acts through the RNAIII effector molecule, silences surface adhesins and induces PSM synthesis, interfering with cell–cell interactions and allowing biofilms to disintegrate (Wang et al., 2024).

**Table 2:** Key molecular regulators of biofilm maturation in *S. aureus.*

Gene/regulator	Function/type	Target genes/pathways	Microbiological role	Effect on biofilm maturation	References
*sarA*	Transcriptional activator	Upregulates *icaADBC*, represses proteases	Enhances PIA production, promotes matrix stability	Positive regulator of biofilm accumulation	Tormo et al. (2005)
*agr*	Quorum-sensing system (RNAIII-mediated)	Downregulates adhesins, upregulates PSMs	Promotes cell dispersal and virulence factor production	Negative regulator of biofilm maturation	Gulba et al. (1989)
*sigB*	Alternative sigma factor	Stress response genes, autolysins	Strengthens the matrix under oxidative/osmotic stress	Enhances biofilm structural integrity	Raman et al. (2001)
*icaR*	*tetR*-family transcriptional repressor	Represses *icaADBC* operon	Modulates PIA synthesis in response to environmental changes	Negative regulator of matrix synthesis	Conlon et al. (2002)
*rsp*	Transcriptional regulator	Downregulates *agr*, modulates virulence	Associated with thicker and more stable biofilms	Indirect positive influence on biofilm formation	Liu and Sun (2020)
*rot*	Repressor of toxins	Balances toxin and adhesin expression	Inhibits dispersal, favors stable biofilm phenotype	Supports mature biofilm maintenance	Mootz et al. (2015)

In response to stress, *sigB*, promotes biofilm maturation by activating genes involved in autolysis, cell envelope integrity, and oxidative stress response mechanisms, thereby reinforcing structural stability. *icaR* is a *tetR*-family repressor that negatively regulates the *icaADBC* operon, finely balancing PIA production in response to environmental pressures (osmolarity, glucose). *Rsp* is a complex regulator (pleiotropy) that inhibits the *agr* system and indirectly favors biofilm. Finally, the *rot* (repressor of toxins) regulates the toxin/adhesin ratio by repressing dispersal-related molecules, thereby contributing to the stability of mature biofilms. These regulators collectively form a highly balanced network of interacting components that specify the architecture, toughness, and tenacity of *S. aureus* biofilms during chronic infection (Valle et al., 2019).

### 2.3 Dispersal mechanisms: role of proteases (SspA, Aur) and nucleases

The last step in the biofilm life cycle of *S. aureus* is dispersion, during which bacteria become planktonic and can further propagate and colonize a new niche when a favorable environment arises. The dispersal process is highly controlled and, in most cases, initiated by stressful environmental factors such as nutrient and oxygen scarcity, acidic pH, and excessive levels of quorum-sensing molecules (Kaplan, 2010). Various enzymes and regulatory networks coordinate dispersal at a molecular level to degrade the biofilm matrix, disrupt intercellular cohesion, and liberate bacterial cells into the surrounding environment. The most essential molecular effectors are proteases, nucleases, and surfactant-like peptides (Beenken et al., 2012). The *sspA* gene encodes a V8 serine protease that digests surface adhesins and matrix-associated proteins, weakening cell–cell and cell-surface adhesions. Correspondingly, the aureolysin (a zinc metalloprotease)-encoding gene *aur* paradoxically synergizes with *sspA* to remodel the biofilm by cleaving structural proteins and increasing its fluidity. The *nuc* gene encodes a thermostable nuclease that degrades eDNA, a key constituent of the biofilm matrix, loosening its cohesion and allowing embedded cells to break free. Collectively, these enzymes serve as degradative effectors that enable biofilms to be dispersed in a well-regulated, environmentally sensitive manner (Kaplan and Horswill, 2024).

Regulatory networks also affect dispersion; e.g., the *agr* quorum-sensing system governs the expression of PSM genes. PSMs exhibit surfactant-like properties, disrupt cell membranes, and enhance the active removal of cells within the biofilm. *Agr*-system is upregulated at the final stages of biofilm growth and is thus thought to be central in triggering dispersal when cells are at high density (Xu et al., 2017). It is observed that the loss or downregulation of dispersal-related genes such as *sspA*, *aur,* or *nuc* results in more resistant, thicker biofilms, leading to a more persistent biofilm that is not affected by antibiotics or host immune clearance. Knowledge of these dispersal mechanisms helps better understand biofilm dynamics and allows exploration of treatment options aimed at stimulating or preventing dispersal to facilitate the containment of the infection (Shree et al., 2023).

### 2.4 Interactions between quorum-sensing (agr) and biofilm development

The *agr* quorum-sensing system is a principal molecular switch that determines the transition between biofilm formation and dispersal in *S. aureus* (Vuong et al., 2000). This system operates through the production, release, and detection of AIPs, which accumulate cell density-dependently and initiate a two-component regulatory cycle involving *agrC* (sensing kinase) and *agrA* (response regulator). Activated *agrA* binds the P2 and P3 promoters, inducing transcription of RNAIII. RNAIII controls many target genes post-transcriptionally, including virulence factor production, cell surface adhesion, and biofilm dispersion (Le and Otto, 2015).

The RNAIII is a multipurpose regulatory RNA, the key effector of the *agr* quorum-sensing system. Unlike a typical regulatory protein, RNAIII primarily regulates gene expression by binding target mRNAs via antisense base pairing, thereby preventing or promoting ribosome binding and altering mRNA stability (Morfeldt et al., 1995; Huntzinger et al., 2005). The mechanism leads to the repression of translation of many surface-associated adhesins and to increased production of extracellular toxins and enzymes, thereby enabling *S. aureus* to switch between colonizing and invasive phenotypes (Novick et al., 1993). Hence, temporal regulation of RNAIII is crucial during biofilm development, because under low agr activity the expression of adhesins that are essential for formation of biofilms is more likely to occur, while an upregulation of the expression of RNAIII during late biofilm maturation stage is expected to increase the production of proteases, nucleases and PSM which are essential for remodeling the matrix and for the dispersal of the bacteria (Wu et al., 2024).

The *agr* regulatory pattern is biphasic during biofilm development. In the early phase of biofilm development, *agr* is rarely expressed, so surface adhesins (*fnbA*, *clfA,* and *spa*) may be expressed to facilitate attachment and intercellular aggregation. At advanced stages of biofilm maturation, the *agr* gene is expressed when bacterial density and environmental pressure rise, adhesin gene expression is suppressed, and dispersal factors are activated. Interestingly, *agr* leads to the expression of PSMs, amphipathic peptides that act as surfactants, causing loss of membrane integrity and cell detachment from the biofilm matrix. At the same time, the matrix is broken and cells released by *agr*-mediated activation of proteases (SspA, Aur) and nuclease assist in preparing *S. aureus* cells to migrate and colonize new locations (Archer et al., 2011). Dynamic control of the architecture by the *agr* system may occur, as it is essential to the *S. aureus* lifecycle and a key to survival across microenvironments. Remarkably, *agr* incompetence or mutations, which are prevalent in clinical isolates of chronic infections, increase biofilm formation, decrease virulence, and increase persistence, thereby reflecting an evolutionary trade-off toward long-term colonization tenure rather than acute pathogenicity (Li et al., 2016). These findings highlight the potential of focusing on the *agr* system to develop new therapeutic strategies to control how the biofilm responds and to fight chronic *S. aureus* infections (Yarwood and Schlievert, 2003).

There is significant cross-talk between *agr* and other global regulators to further refine biofilm regulation. SarA inhibits extracellular proteases while promoting the production of adhesins, thereby reducing the bacterium’s ability to disperse via the *agr*-system and helping maintain the stability of the biofilm matrix (Cheung et al., 2008). In contrast, sigB is a repressor of *agr* activity in response to environmental stress conditions and an inducer of stress response genes that favor persistence under nutrient-poor and/or oxidative stress conditions (Bischoff et al., 2004). This network is further integrated by two regulatory systems that incorporate host-derived signals. ArlRS is involved in maintaining the biofilm structure, either by inducing the expression of adhesins or by repressing early dispersion, while SaeRS regulates the expression of virulence factors, including the nuclease and some extracellular proteases (Burgui et al., 2018; Crosby et al., 2020). SaeRS is not only a virulence regulator but also responsible for biofilm remodeling by regulating the localized degradation of eDNA, conferring structural heterogeneity to mature biofilms and allowing control of their dispersal. These all work together to create the spatial and physiological heterogeneity found in mature *S. aureus* biofilms (Collins et al., 2020; Figure 1).

**Figure 1:**
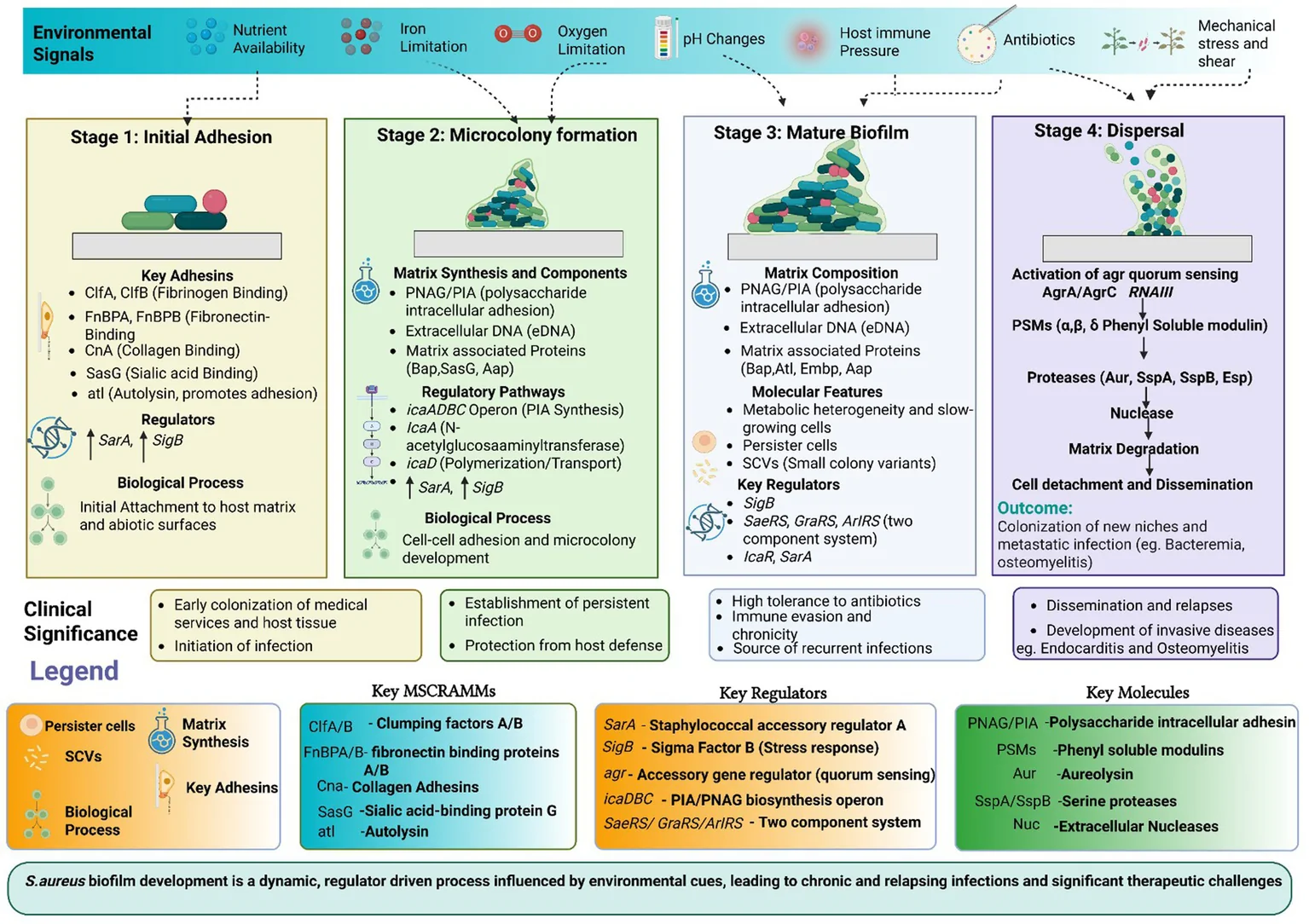
Molecular regulation of *S. aureus* biofilm development, which is stage-specific. The figure shows the key molecular mechanisms involved in the different stages of biofilm development: (i) initial adhesion, (ii) microcolony formation, (iii) biofilm maturation, and (iv) biofilm dispersal. MSCRAMMs, such as ClfA, ClfB, FnbA/B, collagen adhesin (Cna), sasG, and atl, are involved in initial attachment. Biofilm formation relies on the production of PNAG/PIA, eDNA and matrix-associated proteins, which is regulated by the two regulators, sarA and sigB, and *icaADBC*. A mature biofilm consists of an ECM, metabolic heterogeneity, persister cells, and stress-response pathways involving SigB and several TCSs. The *agr*-quorum-sensing system is activated during dispersal, leading to the expression of RNAIII, PSMs, extracellular proteases, and a nuclease, which degrade the matrix, spread bacteria, and establish secondary sites of infection (Queck et al., 2008). These regulatory networks are dynamically regulated during biofilm development in response to environmental signals, including nutrient availability, oxygen tension, pH, iron limitation, host immune pressure, and exposure to antibiotics.

### 2.5 Small regulatory RNAs in biofilm regulation

Small regulatory RNAs (sRNAs) are also important regulators of gene expression that rapidly respond to environmental changes and are important for *S. aureus* biofilm development (Manna et al., 2022; Jones et al., 2024). Most of these sRNAs act via base pairing to modify the accessibility of target mRNAs or the stability of transcripts. The best-characterized example is RNAIII, the main effector of the *agr* quorum-sensing system, which represses several surface adhesins and enhances the expression of toxins, proteases, nucleases, and PSMs (Novick et al., 1993; Boisset et al., 2007). There is a direct link between this regulatory switch and a dispersal-prone biofilm phenotype: strains with high RNAIII expression tend to form thinner, less cohesive biofilms and are more prone to detachment and dissemination, whereas strains with low RNAIII expression prone to form thicker, more persistent biofilms with greater ECM and adhesins (Coelho et al., 2008). Therefore, RNAIII is not only considered a quorum-sensing effector, but a factor that affects the switch between stable sessile growth and invasive planktonic growth.

In addition to RNAIII, several other sRNAs are associated with specific biofilm-related phenotypes. Thus, increased activity of RsaA stimulates surface-associated growth by repressing the global regulator MgrA, which normally inhibits biofilm formation, and this increased activity correlates with increased biofilm biomass, increased PNAG accumulation, and improved persistence under oxidative and nutrient stress conditions (Romilly et al., 2014; Ha et al., 2025). Similarly, RsaE plays a role in metabolic adaptation under oxygen deprivation, which is associated with the phenotype of mature biofilm sub-populations within the inner layers of the biofilm, where oxygen levels are limited (Geissmann et al., 2009). Transcriptomic analysis has also revealed that SprX and its relatives, Spr-family sRNAs, are regulators of cell envelope homeostasis and increased antibiotic tolerance, with changes in their expression linked to greater tolerance to cell-wall-active antibiotics and longer survival of cells within biofilms (Eyraud et al., 2014). A recent study suggests that sRNAs not only regulate the matrix but also contribute to the formation of metabolically diverse and antibiotic-tolerant populations characteristic of *S. aureus* chronic biofilms (Jones et al., 2024).

A new phenomenon gaining significance is the idea that sRNAs act as temporal regulators of biofilm development. Early in the attachment, sRNAs preferentially translate adhesin- and matrix-associated transcripts, thereby facilitating irreversible surface colonization. Nutrient limitation, oxidative stress, and cell-density signals activate a distinct sRNA expression profile that leads to stress adaptation, metabolic dormancy, ECM remodeling, and selective dispersal (Romilly et al., 2014). Previously unknown sRNAs have been identified in high-throughput RNA sequencing analyses of developing biofilms, with expression levels differing among the early attachment, growth, and dispersal stages, indicating that post-transcriptional regulation may be coupled to the *sarA*–*sigB*–*agr* regulatory network (Tomlinson et al., 2021). All these observations are consistent with the idea that sRNAs play direct roles in clinically relevant biofilm phenotypes, such as thick matrix-forming biofilms, stress-resistant mature biofilms, enrichment of persister-cell subpopulations, and dispersal-competent populations, and suggest that sRNAs are promising targets for future antibiofilm therapy in which the bacterial persistence is not the direct target of strong selection pressure (Romilly et al., 2014).

## 3 Regulation of biofilm-associated genes under environmental stress

### 3.1 Impact of pH, glucose, antibiotics, and oxidative stress on gene expression

Inherent genetic factors do not solely determine *S. aureus* biofilm development. Still, it is highly sensitive to environmental *stimuli* that alter the expression of other genes and change the overall structure and functioning of biofilms (Van Roy et al., 2024). pH, glucose level, antibiotic insult, and oxidative stress are critical environmental signals. These environmental signals are actively sensed by global transcriptional regulators and TCS that convert extracellular changes into coordinated transcriptional programs governing adhesion, ECM synthesis, metabolic adaptation, and biofilm dispersal (Crosby et al., 2020).

Chronic wound environments are characterized by acidic pH, which induces and upregulates the *icaADBC* operon, increasing PIA production. It has been reported that under acidic conditions, *S. aureus* induces expression of *sigB* and *sarA* to promote stress adaptation and matrix biosynthesis (François et al., 2023). On the contrary, the alkaline pH prevents biofilm formation by inhibiting adhesin expression and disrupting the matrix structure. The supply of glucose is also essential for biofilm induction. The high glucose levels observed in diabetic patients activate *icaADBC* transcription via *sarA*-dependent mechanisms and repress the *agr*-system, thereby increasing biofilm biomass and persistence (Cendra et al., 2019). Glucose metabolism leads to the accumulation of acetate and a lowered intracellular pH, which strengthens two additional processes: matrix production and the inhibition of biofilm dispersion (Nostro et al., 2012). Another powerful environmental cue affecting biofilm behavior is antibiotic stress. At sub-inhibitory concentrations, antibiotics such as vancomycin, daptomycin, and ciprofloxacin have also been shown to promote biofilm formation by upregulating *atl* and biofilm communities, while selecting for tolerant subpopulations (Kaplan et al., 2012a). At the same time, global regulations such as *sigB* and *rsp* have been initiated to increase resistance to antimicrobial agents and oxidative damage. Host immune cells generate oxidative stress that drives the expression of defense genes, including *katA* (catalase), *sodA* (superoxide dismutase), and DNA repair mechanisms, and favors a robust biofilm by activating a *sarA-* and *sigB*-mediated stress response (Jousselin et al., 2013). All these adaptations allow *S. aureus* to survive within phagocytes and inflamed tissues, thereby aiding immune evasion and contributing to treatment failure (Townsley and Shank, 2017). These points reveal *S. aureus*’s adaptive versatility in response to environmental conditions. The real-time nature of biofilm-related gene regulation ensures survival across various host niches and under high drug pressure. Understanding these environmental signals and their molecular consequences is essential to designing a specific antibiofilm agent that can interfere with biofilm resilience or make target bacterial communities responsive to conventional therapy (Beenken et al., 2004).

Table 3 shows that diverse environmental factors regulate biofilm-related gene expression differently in *S. aureus*, thereby enabling it to adapt and survive in clinical environments. Chronic wounds and abscesses are characterized by an acidic pH (6.0 or lower), which upregulates *sigB*, *sarA*, and *icaADBC*, thereby increasing PIA production and biofilm thickness (Cendra et al., 2019). The presence of high glucose (especially during diabetic infections) suppresses the *agr*-quorum-sensing system, leading to increased *sarA*, *fnbA/B,* and *icaADBC* expression and a strong biofilm structure that is resistant to clearance. Conversely, elevated pH (alkaline, >8.0) and shear stresses, e.g., fluid movement, limit *sarA* and *ica* expression and enhance surface adhesin, e.g., *fnbA,* gene expression to enhance adhesion but disrupt the stability of mature biofilms (Beenken et al., 2004). Exposures to sub-inhibitory concentrations of antibiotics paradoxically induce biofilms by upregulating *atl*, *sarA*, *sigB*, and *icaADBC* and down-regulating *agr*-mediated dispersal, thereby contributing to antimicrobial tolerance. Oxygen availability is another important factor in the physiology of biofilms. When biofilms mature, they develop steep oxygen gradients, and cells in deeper layers become hypoxic and nutrient-limited, leading to decreased respiration and increased fermentative and energy-conserving metabolism (Vlaeminck et al., 2022). The changes in metabolism are also coupled with the activation of SigB-related stress-response pathways and the inhibition of the dispersal function of the *agr*, thereby promoting the maintenance of the sessile lifestyle. As a result, mature biofilms may harbor a variety of spatially distinct cells, some of which may be metabolically active, while other cells at the bottom of the biofilm may be slow-growing or metabolically inactive (Pabst et al., 2016). This physiological heterogeneity can create an environment conducive to the emergence of antibiotic-tolerant persisters and transient small colony variants (SCV) phenotypes. Importantly, hypoxia alone does not necessarily indicate genetic resistance; in the short term, it can reduce antibiotic sensitivity, and once antibiotic pressure is lifted, surviving cells repopulate the biofilm. Additional host-relevant factors, including iron limitation, an anaerobic environment, ethanol exposure, and host proteins such as fibronectin, influence gene expression patterns that favor biofilm survival. This responsiveness of *S. aureus* to environmental factors underscores its complex regulatory plasticity and the difficulty of interfering with its pathogenesis through biofilm production (Azzam et al., 2024).

**Table 3:** Environmental factors influencing biofilm-associated gene expression in *S. aureus.*

Environmental signal	Mechanistic regulatory response	Affected genes/pathways	Resulting biofilm phenotype	References
Acidic pH (≤6.0)	Acidic conditions activate the stress-response regulator SigB, which enhances sarA activity and stimulates *icaADBC* transcription while suppressing agr-mediated dispersal	*↑ sigB, ↑ sarA, ↑ icaADBC, ↓ agr*	Increased PNAG synthesis, thicker biofilm matrix, enhanced structural stability and persistence	Lauderdale et al. (2009)
High Glucose (≥0.5%)	Increased carbon flux promotes SarA-dependent activation of matrix synthesis and represses *agr*- quorum-sensing, favoring biofilm maturation	*↑ sarA, ↑ icaADBC, ↑ fnbA/B, ↓ agr*	Increased adhesion, matrix accumulation and persistent biofilm formation	O’Neill et al. (2008)
Oxygen limitation (Hypoxia)	Restricts respiration and shifts metabolism toward fermentative/energy-conserving pathways; activates SigB-mediated stress adaptation and reduces agr-dependent dispersal	↑ *sigB*, ↓ *agr*, anaerobic metabolism genes	Spatially heterogeneous biofilm with slow-growing, metabolically quiescent and persister-enriched subpopulations; increased antibiotic tolerance and potential emergence of transient SCV phenotypes	Pabst et al. (2016); Yamazaki et al. (2024a)
Oxidative stress (ROS, H₂O₂)	ROS activate oxidative stress-response pathways through SigB and antioxidant regulons, enhancing bacterial survival under immune attack	↑ *sigB, ↑ katA, ↑ sodA, ↑ sarA*	Enhanced oxidative stress resistance and prolonged biofilm persistence	Cosgrove et al. (2007); Painter et al. (2015)
Sub-inhibitory antibiotic exposure	Antibiotic stress activates global stress regulators and autolysis pathways while suppressing agr-dependent dispersal, stimulating matrix production.	↑ *sigB, ↑ sarA, ↑ atl, ↑ icaADBC, ↓ agr*	Increased biofilm biomass and antibiotic tolerance	Schilcher et al. (2016)
Iron limitation	Iron starvation activates iron acquisition systems together with stress regulators that promote biofilm maintenance under nutritional immunity	↑ *isd* genes, ↑ *fur*, ↑ *sarA*, ↑ *sigB*	Enhanced persistence during host-imposed iron restriction	Becker and Skaar (2014)
Anaerobic conditions	Enhanced fermentation and matrix gene expression	↑ *icaADBC*, ↑ *sarA*, ↓ *agr*	Increased biofilm accumulation in low-oxygen niches (e.g., abscesses)	Mashruwala et al. (2017)
Ethanol exposure (1–2%)	Ethanol induces general stress signaling via SigB, increasing expression of matrix-associated genes and reinforcing biofilm architecture	↑ *sigB*, ↑ *sarA*, ↑ *icaADBC*	Accelerated biofilm maturation and increased biomass	Luther et al. (2015)
Shear stress/Flow	Mechanical forces stimulate expression of adhesins while suppressing premature dispersal through reduced Agr activity, strengthening surface attachment	↑ *fnbA*, ↑ *clfA*, ↓ *agr*, ↑ *sarA*	Stronger attachment to biomaterials and vascular surfaces	Pace et al. (2006)
Host-derived ECM proteins (fibronectin, fibrinogen, collagen)	Host ligand recognition induces expression of MSCRAMMs and enhances ArlRS-mediated adhesion programs that initiate stable biofilm formation	↑ *fnbA/B*, ↑ *clfA/B*, ↑ *spa*, ↑ *cna*	Rapid surface colonization and irreversible attachment	Atshan et al. (2021)

Although environmental factors are talked about separately, they act on a relatively small number of regulatory circuits that govern whether *S. aureus* is adhesive, persistent, or dispersive. For instance, nutrient limitation and acidic pH activate the stress-response alternative sigma factor, which induces expression of *sarA*, *icaADBC*, and oxidative stress defense genes and inhibits dispersal through the agr pathways (Valle et al., 2003). The higher the level of SarA activity, the more stable the biofilm becomes, because it down-regulates extracellular protease production and maintains surface adhesin and PNAG production (Lauderdale et al., 2009; Mrak et al., 2012). Acidic conditions, a hallmark of chronic wounds, thus promote thick, tightly structured biofilms that are more resistant to antibiotic killing and host immune clearance. However, under conditions that make nutrients available or when environmental stress is alleviated, agr activity increases, resulting in the expression of *agr* RNAIII and the induction of proteases, nucleases, and PSMs that remodel the ECM and initiate biofilm dispersal (Boles and Horswill, 2008).

### 3.2 Regulatory pathways (e.g., TCSs: ArlRS, SaeRS)

TCSs in *S. aureus* play a significant role in adaptation and environmental signal transduction, enabling the bacterium to promptly change its behavior in response to altered external conditions, including nutrient availability, osmolarity, oxidative stress, and host immune factors (Haag and Bagnoli, 2017). *ArlRS* (autolysis-related locus regulator) and *SaeRS* (*S. aureus* exoprotein expression regulator) are the most critical ones affecting biofilm formation and virulence out of the 16 known TCSs in *S. aureus* (Bleul et al., 2021). These TCSs act through a traditional histidine-aspartate phosphorelase: a membrane-bound sensor kinase (*ArlS* or *SaeS*) binds environmental signals and autophosphorylates, transferring the phosphate to its cytoplasmic response regulator (*ArlR* or *SaeR*), which then regulates subsequent gene expression (Bleul et al., 2021). ArlRS and SaeRS are not single-acting sensors but rather multiple host-derived signals, thereby generating a coordinated transcriptional response. ArlRS specifically responds to changes in surface attachment, osmotic stress, and nutritional conditions, and promotes adhesin expression while inhibiting premature dispersion, thereby promoting biofilm formation (Párraga Solórzano et al., 2019; Crosby et al., 2020). In contrast, SaeRS is specifically induced by host immune signals, including antimicrobial peptides and reactive oxygen species (ROS) produced by neutrophils, leading to context-dependent activation of virulence factors, nucleases, and proteases. This regulatory unit enables *S. aureus* to continually adapt the biofilm’s structure to the microenvironment and to maintain a balance between persistence and dissemination (Mashruwala et al., 2017).

The *ArlRS* system has a dual purpose in *S. aureus* physiology, where it suppresses biofilm and autolysis levels. It has been demonstrated to exert positive control over the expression of surface adhesins, e.g., FnbA and ClfA, and to negatively regulate the expression of biofilm-degrading proteases (Crosby et al., 2020). *ArlRS*, by this rule, can increase initial attachment and stimulate the development of stable, structured biofilms. *ArlRS* also suppresses the *agr*-quorum-sensing system, which is pro-dispersal and favors long-term colonization of surfaces, a trait of particular clinical importance in device-associated infections, where prolonged biofilm survival can be problematic (Walker et al., 2013). Mutants lacking *ArlRS* show reduced biofilm biomass and impaired adhesion; thus, this role as a positive sessile growth regulator is confirmed (Crosby et al., 2020).

The *SaeRS* system, on the other hand, is more responsive to host-derived signals, such as ROS and antimicrobial peptides produced by neutrophils, and it controls a repertoire of virulence factors, including *hla* (alpha-toxin), *sspA* (serine protease), and *nuc*. Although it is conventional to characterize acute virulence, *SaeRS* plays a role in biofilm sensing, influencing matrix composition and the expression of dispersal effectors. In late-stage biofilm development or under immune pressure, *SaeRS* can activate *nuc* and *sspA*, leading to partial breakdown and cell dispersion. Amazingly, *SaeRS’s* energy-dependent lifestyle is closely linked with *agr*, with its output being a context-specific biofilm, either sustainable or regulated detachment (Butrico and Cassat, 2020). This makes the character of crosstalk among TCSs and global regulators very complex in *S. aureus*. It allows it to better control its biofilm phenotype in response to environmental signals, facilitating its survival during infection and antimicrobial drug treatment.

Recent studies have also shown that biofilm development is not uniform, with genetically programmed metabolic heterogeneity. Mature biofilms contain distinct subpopulations that have differences in respiratory activity, metabolic flux, stress responses, and expression of matrix-remodeling enzymes (Selvam et al., 2026). The regulatory mechanism of stochastic nuclease expression results in localized degradation of eDNA, channel formation, and structural heterogeneity, thereby increasing nutrient diffusion and allowing the dispersion of the selected bacterial subpopulation (DelMain et al., 2020). This phenotypic diversification enables the biofilm to maintain persistence over time as oxygen levels, nutrient availability, and exposure to antimicrobials change.

### 3.3 Transcriptomic and proteomic changes during biofilm maturation

Reprogramming of the transcriptome and proteome during biofilm maturation in *S. aureus* facilitates this switch, as bacterial populations shift to growth via non-divisive states, exhibit highly diverse metabolisms, and can resist stresses (Resch et al., 2005). RNA-seq transcriptomic studies have found that during maturation, many genes associated with ECM production, including the *icaADBC* operon, the PNAG output, SarA, which positively regulates matrix deposition, and negative extracellular protease genes, were significantly upregulated (Tomlinson et al., 2021). Simultaneously, there is downregulation of *agr* activity, resulting in decreased expression of dispersal factors associated with *psm* genes and reduced toxin production via RNAIII (DelMain et al., 2020). This transcriptional change is an ode to a commitment to a sessile biofilm, its retentive phenotype (Vlaeminck et al., 2022). Likewise, regulators of stress responses, e.g., *sigB,* are activated, leading to the expression of genes that confer protection against oxidative stress, promote thickening of the peptidoglycan layer, and promote survival in the stationary phase.

More recently, transcriptomic investigations have also shown that biofilm development is not a series of sequential regulatory events but a dynamic process (Aboelnaga et al., 2024). The expression of key regulators of biofilm formation has been shown to vary continuously during biofilm growth and across different *S. aureus* lineages, as revealed by longitudinal RNA-sequencing analysis (Malachowa and DeLeo, 2025). In early attachment, adhesin genes such as *fnbA/B and clfA/B,* as well as matrix-associated genes, are preferentially expressed to establish surface colonization. As the biofilm ages, transcription gradually shifts toward stress adaptation, anaerobic metabolism, ECM maintenance, and nutrient acquisition, while expression of global regulators such as *sarA* and *sigB* increases. At the end of maturation and dispersal, proteases, nucleases, and PSM are expressed in response to activation of the *agr* regulon, thereby remodeling and dispersing the biofilm (Resch et al., 2005). The results show that, within the same regulatory genes, temporal transcriptional reprogramming underlies biofilm regulation rather than the constant expression of a “set repertoire” of genes.

Proteomic biofilm maturation includes a greater abundance of structural and functional components of the matrix [surface adhesins (FnbA/B, ClfA), Atl, and matrix-stabilizing enzymes (IsaA, SsaA)]. Enzymes involved in anaerobic metabolism (e.g., lactate dehydrogenase, alcohol dehydrogenase) are also overexpressed in proteomic profiling, as oxygen-restricted microenvironments characterize mature biofilms (Freiberg et al., 2016). Proteins with antioxidant properties, including KatA and SodA, are enriched, providing stronger defense against ROS and DNA damage (Patlevič et al., 2016). Translational-related proteins and the ribosomal subunits are specifically found to be depressed, consistent with an increase in metabolically inactive sub-population in the deeper layers of the biofilm. This metabolic diversity promotes *S. aureus* survival in unfavorable environments and contributes to antibiotic tolerance, immunity, and chronic infection (Ali et al., 2025).

In summary, transcriptomic and proteomic analyses show that biofilm development is a dynamic developmental program, in which the activation of the same regulatory genes is repeated, while the activity of others is continuously reorganized (Tomlinson et al., 2021). While the main regulators, *sarA*, *agr*, *sigB*, and *icaADBC* are needed throughout biofilm development, their downstream targets, interactions with each other, and their biological roles evolve significantly depending on the age of the biofilm, its metabolic state, and its environmental context. The systems-level view of *S. aureus* biofilms reveals their tremendous adaptability and supports targeting temporal regulatory networks as future antibiofilm drug targets rather than single genes.

Importantly, the metabolically diverse organization affords the physiological conditions under which reversible persister states and SCV phenotypes arise, and connects the growth of an antibiotic-tolerant biofilm to the ability of surviving populations to mediate chronic infection.

### 3.4 Persister cells and SCVs: metabolic adaptation and antibiotic tolerance

Biofilms of mature *S. aureus* consist of physiologically heterogeneous subpopulations that can be in low growth or metabolically altered states depending on nutrient availability, hypoxia, oxidative stress, acidic pH, and antibiotics (Table 3; Vlaeminck et al., 2022). The gradients of oxygen and nutrients within the biofilm reduce respiratory activity and shift carbon metabolism toward fermentative processes and energy conservation in deeper parts of the biofilm. Along with the activation of the stress-response regulator sigB, these conditions, alongwith the repression of dispersal via the *agr* gene, create an environment of stress response, decreased growth, and antimicrobial resistance (Lauderdale et al., 2009). Experiments with biofilm models show that oxygen limitation leads to metabolic heterogeneity and antibiotic resistance, thereby creating a physiological environment conducive to the enrichment of tolerant subpopulations (Choi and Kim, 2024).

However, persister cells and SCVs need to be differentiated mechanistically. Persisters are phenotypic, reversible states in which a subpopulation of genetically susceptible bacteria temporarily switches to a growth-arrested or slow-growing state and survives antibiotic exposure without acquiring heritable antimicrobial resistance. In the absence of stress, persisters can recover and revert to the parental phenotype (Liu et al., 2024). In *S. aureus*, persister cells are characterized by decreased ATP levels, metabolic changes, altered protein homeostasis, and activation of stress response pathways. SCVs, on the other hand, are a colony and physiological phenotype characterized by significantly decreased growth, altered metabolism, and increased persistence (Liu et al., 2024). Some SCVs are transient and reversible, whereas stable SCVs can result from heritable mutations in metabolic pathways, including electron transport and heme or menadione biosynthesis (Dean et al., 2014). Therefore, it is important not to consider the SCV synonymous with persisters: phenotypically induced SCVs can overlap with persisters, whereas genetically stable SCVs represent a heritable state.

While sigB has been shown to be a direct link in the chain of environmental stress, biofilm maturation, and the SCV phenotype, it also represents an essential regulatory step in this pathway. A chronic sigB response leads to stress adaptation, changes in the expression of adhesins and ECM factors, and may promote biofilm growth and the persistence of bacteria within the host (Wang et al., 2022). Experimental studies have demonstrated that sigB is involved in the emergence of SCV after aminoglycoside exposure, in the formation of SCV-associated biofilms, and that sigB activity is crucial for the persistence phenotype of clinical SCV strains (Mitchell et al., 2010). The results suggest that persistent phenotypes could be produced without a stable genetic change in a stress-induced fashion.

Thus, hypoxia, nutrient limitation, changes in central metabolism, activation of stress responses, and decreased cell proliferation combine to create a protected reservoir of antibiotic-tolerant cells in mature biofilms. Importantly, conventional antibiotics are most bactericidal against metabolically active, dividing cells, so that persisters and metabolically quiescent SCVs may persist at bactericidal antibiotic concentrations (Freiberg et al., 2024). The cells that survive the antimicrobial pressure can grow back after the pressure is removed, repopulate the biofilm, and lead to recurrent infection. This offers a mechanistic link between physiological heterogeneity in biofilms and the persistence of *S. aureus* infections, especially in chronic infections such as osteomyelitis, prosthetic device infections, and other chronic infections where repeated antimicrobial exposure can drive the selection for or enrichment of tolerant subpopulations (Sivori et al., 2024).

## 4 Role of biofilm-forming genes in chronic and device-associated infections

### 4.1 Biofilm in endovascular, bone, and soft tissue infections

All the above regulatory mechanisms ultimately manifest as clinically relevant phenotypes during chronic infection. The biofilm-associated regulators are not distinct genetic pathways; rather, their activation and repression within their temporal context determine *S. aureus*’s ability to adapt to various host microsites during infection (Sivori et al., 2022). Therefore, knowledge of stage-specific transcriptional programs provides greater insight into biofilm persistence than knowledge of individual genes.

Staphylococcal biofilms are central to the pathogenesis of chronic, recurrent infections in endovascular, and soft-tissue compartments. These infections are often resistant to conventional antimicrobial interventions because biofilm-associated cells exhibit metabolic changes, increased stress responsiveness, and dense extracellular matrices that inhibit antibiotic uptake (Di Domenico et al., 2022). In infective endocarditis or catheter-blood infections, *S. aureus* binds endothelial cells of the vessel or non-biotic surfaces via adhesins, including ClfA, ClfB, and FnBPs. These microbial surface components, which are MSCRAMMs, can interact strongly with host plasma proteins, including fibrinogen, fibronectin, and von Willebrand factor (Claes et al., 2014). The shear stress of blood flow also enhances biofilm growth by increasing the expression of adhesins and downregulating genes involved in dispersion, such as *agr,* thereby allowing cells to remain on intravascular devices and valves (Bhattacharya and Horswill, 2024). *S. aureus* biofilms dominate infections of cortical bone canaliculi (osteomyelitis) and implanted orthopedic devices, and are resistant to both antibiotics and the host defense. Pathogens interact with their Cna and FnBPs to attach to bone tissue proteins and trigger biofilm development in osseous tissue.

*S. aureus* responds to the nutrient and oxygen-poor bone microenvironment with metabolic reprogramming and metabolic shifts to less respiration and more fermentation, followed by activation of stress-adaptation pathways, including sigB (Potter et al., 2020). These conditions can select for slow-growing, antibiotic-tolerant persister populations and/or for transient or stable SCV phenotypes. Transient SCV-like states may be physiological adaptations that are reversible, whereas stable SCVs can be inherited defects in metabolic pathways, resulting in intracellular persistence and antibiotic resistance (Bollar et al., 2024). The simultaneous presence of these reversible and stable phenotypes in bacteria from biofilm-associated osteomyelitis suggests a potential cause for the chronic and relapsing nature of infection: a reservoir of viable bacteria that persists through antimicrobial therapy.

Biofilm formation also strongly affects soft tissue infections, including diabetic foot ulcers, surgical wounds, and abscesses. Biofilms protect the bacteria in these niches against phagocytosis by host and inflammatory cells, allowing long-lasting inflammation and tissue damage. Chronic wounds thus have a permissive environment characterized by acidic pH, elevated glucose levels, and poor perfusion, which promotes biofilm growth. In this case, *S. aureus* induces *icaADBC* expression to produce PNAG and inhibits *agr-*mediated dispersion to sustain a sessile phenotype (Guzmán-Soto et al., 2021). Furthermore, when other organisms are co-infecting (e.g., *Pseudomonas aeruginosa*), the biofilm can be more stable, employing interspecies signaling and sharing the biofilm, making it more difficult to manage. The ability to survive oxidative and immune pressure has been supported by proteomic analysis showing increased levels of detoxifying proteins, including KatA and SodA. At these discrete anatomical locations, *S. aureus* leverages environmental conditions and host-tissue interactions to tune gene expression and maximize biofilm robustness (Al-Wrafy et al., 2023). Along with reversible metabolic dormancy and, in some cases, heritable metabolic mutations that generate stable SCV phenotypes, these site-adapted biofilm states promote immune evasion, antibiotic tolerance, chronic colonization, and recurrent infection. Knowledge of the molecular basis of biofilm phenotypes in these clinically significant infections may inform the development of specific therapeutic interventions, e.g., anti-adhesin agents, quorum-sensing inhibitors, or enzymatic disruption of biofilms to enhance clinical outcomes (Uruén et al., 2020).

A comparison of the molecular signatures of *S. aureus* biofilm formation at different clinical sites is summarized in Figure 2. Although conserved biofilm regulators (*icaADBC*, *agr*, *sarA*, *sigB*) are shared across infections, biofilm components (adhesins, virulence factors, immune evasion strategies, clinical impact) vary by the infected tissue or implanted medical device. This demonstrates the context-dependent adaptation of *S. aureus* biofilms along disease progression (Beenken and Smeltzer, 2025).

**Figure 2:**
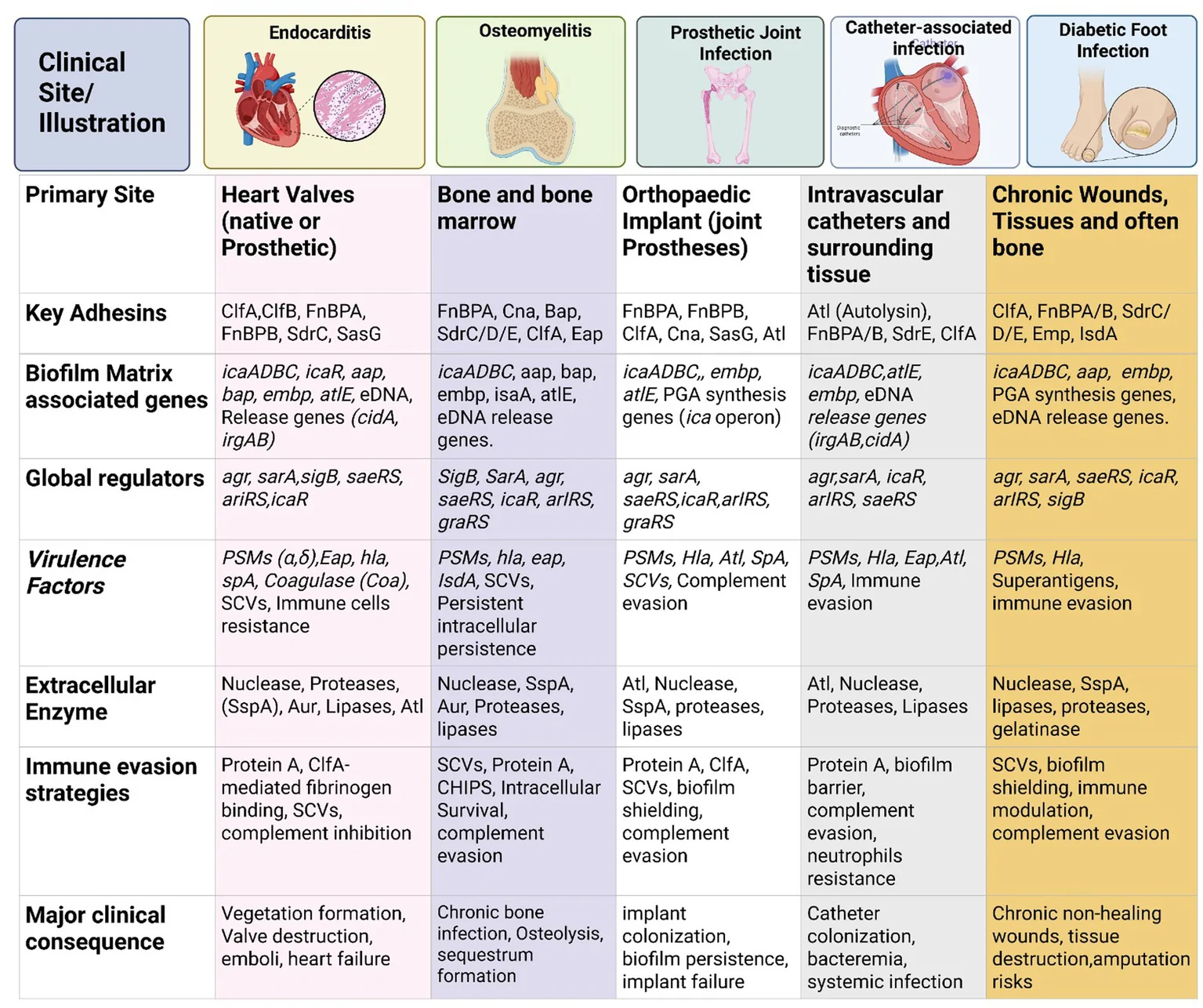
Molecular determinants and clinical manifestations of *S. aureus* biofilm-associated infections in key infection locations. The figure summarizes the site-specific molecular signature of *S. aureus* biofilm-associated infections, including infective endocarditis, osteomyelitis, prosthetic joint infection, catheter-associated infection, and diabetic foot infection. It emphasizes the most common adhesins, genes involved in the biofilm matrix, global regulatory networks, virulence factors, ECM, immune evasion mechanisms, and clinical outcomes for each infection type, as well as how biofilm composition relates to tissue-specific pathogenesis and persistence.

### 4.2 Clinical studies showing gene prevalence in chronic infections

Other chronic infections with *S. aureus,* such as prosthetic joint infection, diabetic foot ulcer, osteomyelitis, and less frequent endocarditis, are increasingly linked to biofilm formation and overexpression of specific genetic determinants (Afonso et al., 2021). Numerous clinical investigations have revealed that isolates from these chronic environments often harbor biofilm-related genes, particularly those encoding adhesion, matrix synthesis, and communication (Mamdoh et al., 2023). While *icaADBC* is commonly found in clinical isolates that grow in a biofilm, the presence of *icaADBC* does not necessarily indicate PNAG dependence, as certain clinical isolates of *S. aureus* are able to produce protein- and/or eDNA-dominant biofilms without *icaADBC* (Yu et al., 2023). A meta-analysis of orthopedic implant infections indicated that more than 70% of *S. aureus* isolates were *ica*-positive, with strong associations with large biofilm biomasses and relapse rates. The results suggest that *icaADBC* is actively expressed in the chronic infection niche (Hussain et al., 2017).

Surface adhesin genes, including *fnbA*, *fnbB*, *clfA*, *clfB,* and *icaADBC,* are present in clinical isolates of non-healing wounds and bloodstream infections. These genes encode MSCRAMM, which explains how these bacteria bind strongly to host matrix proteins and biomaterial surfaces early in infection. *S. aureus* isolates obtained in a large-scale study aiming to collect *S. aureus* isolates in diabetic foot ulcers showed that 82% contained the gene *fnbA* and 76% possessed the gene *clfA*, both of which were highly linked with the development of increased biofilm-forming capacity and multidrug resistance (Ghasemian et al., 2015). Remarkably, strains co-expressing *fnbA* and *icaA* exhibited thicker biofilms and increased tolerance (or reduced susceptibility) to vancomycin and rifampicin. Global transcription factors controlling these adhesin genes play a key role in chronic infection persistence, with *sarA* being overexpressed in high-biofilm-forming clinical isolates (Jin et al., 2020).

The widespread repression or mutation of the *agr* quorum-sensing system, which would generally lead to dispersal and toxin production, is evident in further molecular profiling of clinical isolates (Schilcher and Horswill, 2020). Isolates of chronic rhinosinusitis, osteomyelitis, and prosthetic valve endocarditis with dysfunctional *agr* variants have been identified as preferentially promoting sessile growth with immune avoidance rather than cytotoxicity. An investigation involving *S. aureus* bloodstream isolates from patients with prolonged bacteremia found that strains with *agr* dysfunction displayed significantly elevated biofilm formation, which was associated with poor clinical outcomes. These are frequently associated with increased expression, suggesting a compensatory increase in biofilm stabilization and stress resistance (Douiyeb et al., 2025).

Taken together, these observations suggest that the ability to form strong biofilms is linked to multiple overlapping yet distinct genetic programs in persistent *S. aureus* infection, rather than to a single matrix pathway. Biofilm production in many isolates depends on icaADBC/PNAG, but proteins via icaADBC-mediated mechanisms, such as FnbA/B and sasG, and other surface-associated adhesins can also contribute to robust biofilm production without icaADBC/PNAG (Hernández-Cuellar et al., 2023). The proportions of the different matrix components may vary with strain background, infection place, and environmental conditions, indicating that molecular diagnostics and antibiofilm therapy should account for matrix composition rather than assume biofilm structure based solely on the presence of *icaADBC* (Rudolph et al., 2026).

Such molecular adaptations are necessary for long-term colonization and are associated with enhanced antibiotic tolerance, immune resistance, and relapse rates after therapy. Combining molecular diagnostics to identify these gene expression profiles may significantly increase the predictive value of biofilm-related pathology and rational selection of antibiofilm therapies in clinical settings (Azmi et al., 2019).

Figure 3 outlines the genetic and environmental control of *S. aureus* biofilm development. MSCRAMM genes (*fnbA/B*, *clfA/B*, *cna*) mediate surface binding in the adhesion phase. Maturation involves ECM production via *icaADBC* and regulation by *sarA*, *sigB*, and *icaR*. Environmental conditions—high glucose, acidic pH, and oxidative stress—modulate biofilm stability and dispersal readiness. Dispersal is triggered by *agr*-mediated activation of enzymes that degrade the matrix. This integrated network ensures biofilm persistence while allowing timely release of planktonic cells to colonize new sites (Cerca et al., 2008).

**Figure 3:**
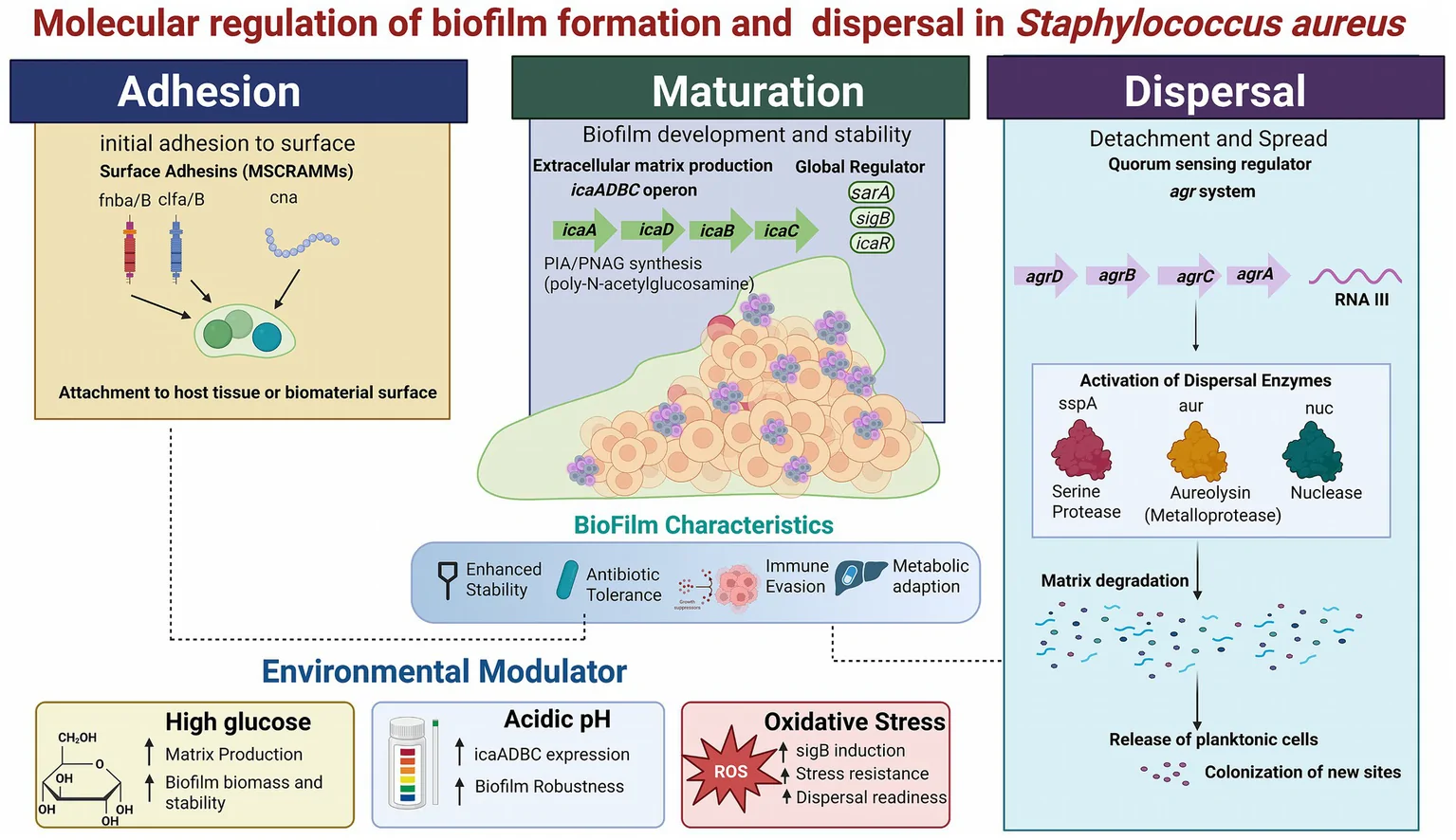
Genetic and molecular regulation of biofilm formation & dispersal in *S. aureus*. The three major phases of *S. aureus* biofilm development—Adhesion, Maturation, and Dispersal—along with their key genetic regulators and environmental modulators. Adhesion is driven by surface adhesin genes (*fnbA/B*, *clfA/B*, *cna*), followed by maturation, regulated by matrix synthesis and global control genes (*icaADBC*, *sarA*, *sigB*, *icaR*). Environmental factors such as glucose, pH, and oxidative stress influence maturation and dispersal. The dispersal stage involves enzymatic degradation via *sspA* (serine protease), *aur* (aureolysin), and *nuc* (nuclease), primarily coordinated by the *agr* quorum-sensing system. Arrows indicate the progression of biofilm stages and regulatory connections to dispersal effectors.

### 4.3 Differences in gene expression between planktonic and biofilm forms *in vivo*

Previous research has shown significant differences in gene expression between biofilm-associated and planktonic cells in *S. aureus* (Vlaeminck et al., 2022). Planktonic cells exhibit higher expression of acute virulence genes than biofilm cells. In contrast, biofilm-resident bacteria exhibit transcriptional compaction that promotes adhesion, matrix formation, and immune evasion. Biofilm cells also downregulate quorum-sensing systems, e.g., *agr,* and upregulate regulators, e.g., *sarA*, *sigB,* and *r*sp., which, in turn, activate genes encoding adhesins (*fnbA*, *clfA,* and *spa*) and matrix (*icaADBC*) components. These are adaptations aiming at the sessile lifestyle, survival in hostile host conditions, and resistance to antimicrobials (Secor et al., 2011). Transcriptomic experiments in animal models and human infection systems have demonstrated that biofilm cells exhibit heightened expression of disease stress response genes (e.g., *katA*, *sodA*, *recA*) and reduced expression of energy-intensive biosynthetic and translational machinery (Slany et al., 2017). Such a state of metabolic quiescence also contributes to phenotypic antibiotic tolerance and helps survive the chronic infections’ inflamed or nutrient-poor microenvironment. By contrast, planktonic cells depend more on glycolysis and the TCA cycle for growth and virulence factor secretion; thus, they are more vulnerable to the immune response and antimicrobials. These changes in gene expression result in a significant phenotypic transition, enabling *S. aureus* to switch between acute and chronic infection stages (Caldara et al., 2025).

Table 4 reflects significant differences in gene expression between planktonic and biofilm-associated *S. aureus* cells *in vivo,* demonstrating adaptation of these forms to distinct physiological habitats. The expression of the *agr*, *psm*, and *hla* genes are strongly upregulated in planktonic cells that it leads to toxin release, immune induction, and bacterial dissemination, characteristics of acute virulence (Berube et al., 2014). Biofilm cells, on the other hand, are characterized by a striking suppression of these virulence pathways and elevated induction of global regulators, which in turn upregulate matrix biosynthesis genes (e.g., *icaADBC*) and adhesins (e.g., *fnbA*, *clfA*, *spa*), all inimical to biofilm cohesion and chronic infection (Patel and Rawat, 2023). The biofilm cells also exhibit more stress response genes, such as *katA*, *sodA*, and *recA*, that confer resistance to host oxidative and DNA-damaging defenses. Interestingly, ribosomal and TCA cycle genes are downregulated in biofilm cells, reflecting a shift toward metabolic dormancy and anaerobic fermentation, a phenomenon that minimizes antibiotic susceptibility. These transcriptional modifications highlight the phenotypic differences between planktonic and biofilm growth modes, making biofilm-related infections difficult to treat and manage (Volk et al., 2025).

**Table 4:** *In vivo* gene expression differences between planktonic and biofilm forms of *S. aureus*.

Gene/pathway	Planktonic cells	Biofilm cells	Functional implication	References
*agr*, *psm*, *hla*	Highly expressed-promotes toxin secretion, dispersal	Downregulated-promotes stability and immune evasion	Acute virulence *vs*. chronic persistence	Yamazaki et al. (2024b)
*sarA*, *sigB*, *rsp*	Basal expression	Upregulated-enhances adhesins, matrix genes, and stress responses	Promotes biofilm integrity and antibiotic tolerance	Valle et al. (2003)
*icaADBC*	Low or repressed	Strongly upregulated-PIA matrix synthesis	Structural maintenance of biofilm	Shree et al. (2023)
*fnbA*, *clfA*, *spa*	Variable-expressed under adhesion stimuli	Upregulated-promotes surface attachment and immune evasion	Facilitates colonization and chronicity	Stutz et al. (2011)
*katA*, *sodA*, *recA*	Moderate expression-basal oxidative/DNA stress defence	Strongly upregulated-survival in hostile environments	Defence against host ROS and DNA damage	Painter et al. (2015)
Ribosomal genes	Highly expressed-active protein synthesis	Downregulated-metabolic dormancy	Reduces susceptibility to antibiotics targeting active metabolism	Shiraliyev and Orman (2024)
TCA cycle, glycolysis	Upregulated-supports rapid proliferation	Downregulated-favors fermentation, metabolic shift	Energy conservation under nutrient-limited, hypoxic biofilm conditions	Vitko et al. (2015)

## 5 Therapeutic challenges and future strategies

Many antibiofilm strategies have been shown to be effective against *S. aureus*, but they differ in translational maturity. Several methods have been tested *in vitro*, and some have advanced to animal models or early clinical trials. For this reason, the following sections categorize therapeutic approaches by the current state of the evidence, highlighting their advantages, disadvantages, and translational potential. This evidence-based research enables the critical assessment of new therapies and identifies the remaining challenges that must be overcome before it becomes common practice in clinical care.

### 5.1 Mechanism-based antibiofilm therapeutic strategies targeting *S. aureus*

Therapeutic strategies to treat *S. aureus* biofilm-associated infections must be directed at the extracellular biofilm matrix and regulatory pathway that promote biofilm maintenance. Mechanism-based antibiofilm approaches aim to disrupt mature biofilms, enhance antibiotic uptake, and reduce chronic persistence, in contrast to conventional antibiotic treatment, which is primarily geared toward killing metabolically active planktonic bacteria (Kaplan et al., 2012b; Sugimoto et al., 2018; Liu et al., 2021). The ability of matrix-degrading enzymes, such as DNase I, Dispersin B, lysostaphin, and proteolytic enzymes, to directly degrade eDNA, PNAG, and proteinaceous matrix components disrupts biofilm architecture and renders bacteria more susceptible to host immune responses and antimicrobial drugs (Lin et al., 2024). These methods, which primarily disrupt the protective biofilm scaffold, are especially attractive when combined with conventional antibiotics, making them a suitable additional therapeutic strategy.

The second approach aims to disrupt the regulatory networks that coordinate biofilm development. The inhibition of bacterial communication by quorum-sensing inhibitors reduces the expression of virulence factors and PSMs and prevents bacterial dissemination via mature biofilms or dispersal. Likewise, compounds that inhibit sarA or modulate the activities of the sigB-dependent stress response affect matrix production, adhesin expression, and biofilm stability (Periasamy et al., 2012; Zhao et al., 2023). The advantage of these regulatory routes is that they are not as virulent as bacteria and do not impose strong selective pressure, unlike bactericidal antibiotics, which can do so and lead to the development of antimicrobial resistance. With growing insight into post-transcriptional regulation, small regulatory RNAs and TCS such as *ArlRS* and *SaeRS* have become promising therapeutic targets (Kwiecinski et al., 2022; Ha et al., 2025).

Targeted delivery systems and novel antimicrobial modalities are combined to create new options within the therapeutic landscape, extending the reach of emerging technologies. Experimental studies have demonstrated promising antibiofilm activity for antimicrobial peptides, bacteriophages, phage-derived lysins, nitric oxide-releasing systems, and nanoparticle-based delivery platforms through mechanisms that include enhanced biofilm penetration, disruption of the ECM, selective killing of biofilm-associated bacterial populations, and improved delivery of antimicrobial agents (Zhang et al., 2018; Landa et al., 2024; Ponce Benavente et al., 2024; Zhang K. et al., 2024). However, the majority of these methods remain in the preclinical and early translational stages, and factors such as delivery efficiency, biofilm heterogeneity, host compatibility, the definition of clinically meaningful endpoints, and regulatory requirements pose obstacles to widespread clinical use. Given the differences in matrix composition, regulatory state, and conserved target sequences among clinical isolates, a one-size-fits-all antibiofilm intervention is unlikely to be effective, and mechanism- and strain-appropriate combination therapies are better suited for clinical management.

#### 5.1.1 Translational and pharmacokinetic bottlenecks

Although there is promising preclinical activity, translation of other macromolecular agents, such as antibiofilm enzymes, AMRs, and others, into routine clinical use is limited by pharmacokinetic and tissue-delivery issues. Proteolytic degradation, denaturation, renal clearance, and rapid removal from the site of infection account for the relatively short half-lives of many enzymes and peptides *in vivo*. This means that systemic dosing may not achieve sufficient and/or sustained levels at the biofilm, while higher systemic doses may lead to higher toxicity, immunogenicity, or off-target effects. The challenge is especially relevant to matrix-degrading enzymes such as DNase I and Dispersin B, which are therapeutic agents that require critical concentrations at the biofilm surface and for an appropriate duration (Li et al., 2023). Exposure may be extended by encapsulation, protein engineering, or PEGylation, but these modifications can significantly affect the protein’s enzymatic activity, tissue distribution, immunogenicity, and manufacturing complexity.

The other significant challenge is accessing fully developed biofilms. The ECM is spatially heterogeneous and may hinder the diffusion and penetration of therapeutic molecules, and metabolically quiescent cells and/or persisters in deep biofilm areas may be less sensitive to conventional antimicrobial agents (Bin et al., 2025). Hence, homogenous drug levels within the biofilm represent a key pharmacodynamic need. Local delivery of the drug can be achieved through topical delivery to chronic wounds, inhaled delivery for respiratory infections, or antibiotic- or antibiofilm-loaded coatings, hydrogels, and implant-associated delivery systems, which can produce greater local drug exposure with reduced systemic toxicity (Zhang L. et al., 2024). But there are limitations to the local delivery approach, and it can be technically challenging for less common infections, such as prosthetic-device infections, endocarditis, and deep-seated infections like osteomyelitis (Reinhart et al., 2024). On the other hand, systemic administration is useful for infections that are disseminated or not accessible at the anatomical site and require predictable tissue penetration, sustained activity in the body, and an acceptable systemic safety profile (Lodise et al., 2026). Therefore, the pharmacokinetics, penetration into biofilms, and pharmacodynamic activity at the infection site may be necessary, rather than a simple increase in the drug’s systemic level, to achieve successful translation.

#### 5.1.2 Strain-to-strain heterogeneity and target variability

One of the major translational challenges is the variability in *S. aureus* biofilms across strains, which can make a potential antibiofilm target present but either unexpressed or inaccessible to function. There is substantial interclonal variation in biofilm formation, both in the relative importance of PNAG and eDNA *versus* proteinaceous matrix components, and in the activity of global regulators such as *agr*, *sarA*, and *sigB* (Rudolph et al., 2026). This means that PNAG-producing strains capable of forming eDNA- or protein-dependent biofilms may be less susceptible to PNAG-targeting agents, despite expressing *icaADBC*. Comparative transcriptomic analyses of different *S. aureus* lineages have also revealed significant differences in the regulation of response and biofilm development (Alibegli et al., 2025).

An added source of therapeutic heterogeneity is the variation in the *agr* system. *S. aureus* exists in four major allelic groups of *agr*, and sequence polymorphisms in the agrBDCA can have an effect on quorum-sensing activity and downstream virulence and biofilm phenotypes. Furthermore, chronic *agr* activities and/or agr dysfunction are often linked to chronic biofilm status (Pivard et al., 2026). Therefore, the effectiveness of quorum-sensing inhibitors can vary depending on the agr genotype, the regulatory background of the infecting strain, and the strain’s functional status (Polaske et al., 2026). Other therapeutic targets, such as matrix-associated adhesion proteins or sequence-specific targets for CRISPR-based antimicrobials, may also vary in sequence, potentially affecting target recognition or expression. Such observations form the basis for a precision antibiofilm strategy that aims to characterize the matrix composition, *agr* status, target gene-sequence, and associated biofilm genotypes prior to selecting an intervention (Mills et al., 2024).

#### 5.1.3 Difficulty establishing clinically meaningful endpoints for biofilm-targeting therapies

One of the biggest challenges in translating new biofilm therapies into the clinic is the lack of commonly agreed-upon clinical endpoints that directly and reliably assess biofilm disruption and predict clinical benefit. In contrast to traditional antibiotics, which can often be tested for microbiological eradication, clinical cure, or survival, the activity of biofilm-directed agents may most importantly affect the integrity of the biofilm matrix, the spatial structure of the bacteria, the metabolic state of the bacteria, or the susceptibility of the bacteria to the antibiotic (Ponce Benavente et al., 2024; Sun et al., 2025). Furthermore, it is hard to quantify the burden and composition of biofilm over time *in vivo*, especially in deep-seated infections and infections involving prosthetic material. Therefore, a decrease in ECM components, gene expression in the biofilm, or biomass can serve as a pharmacodynamic biomarker, but it is not necessarily the best substitute for a patient-centered outcome (Doub et al., 2024).

Therefore, clinical trial endpoints should aim to separate mechanistic biofilm endpoints from clinically meaningful endpoints. Relevant endpoints can vary based on the infection, including microbiological eradication or sustained clearance, clinical cure, time to symptom resolution, wound healing, device retention or removal, recurrence or relapse, need for surgical intervention, infection-related hospitalization, and mortality (Conway et al., 2024). The lack of microbiological improvement after treatment may be of particular concern in chronic or device-associated infections, as durable response and freedom from recurrence over a sufficiently long follow-up period may be more meaningful. Combining clinical response, microbiological outcome, and recurrence into one or more composite endpoints may be the best available measure (Petropoulou et al., 2025). Future trials should also include validated biomarkers of pharmacodynamic activity that reflect biofilm and correlate these measures to patient-centered outcomes, as a new pharmacodynamic endpoint linked to patient outcomes. To assess the clinical impact of biofilm disruption and to enable inter-study comparisons of antibiofilm trials, standardized endpoint definitions across infections may be key.

### 5.2 Novel antibiofilm strategies

#### 5.2.1 Enzyme-based biofilm disruption (DNase, dispersin B)

One of its major structural elements in *S. aureus* biofilms is eDNA, particularly at the early and intermediate stages of biofilm growth. It is produced during the autolysis of a subpopulation or by active secretion, and it acts as an intercellular scaffold that stiffens the biofilm’s three-dimensional structure. DNase I is a promising agent that functions as a biofilm disruptor, a nuclease that hydrolyzes DNA by cleaving phosphodiester bonds (Kaplan et al., 2012b). Molecular studies have demonstrated that DNase I treatment reduces biofilm by dissolving the eDNA mesh, disrupting sessile bacteria, and promoting a planktonic state, thereby increasing vulnerability to both antibiotics and host-acquired immune responses. Notably, DNase I does not cause cytotoxicity or resistance, and the most effective applications are during the initial phases of biofilm formation or in combination with antibiotics such as vancomycin or rifampicin (Lister and Horswill, 2014).

On a molecular level, DNase I treatment influences regulatory pathways by decreasing eDNA-mediated signaling and stabilizing biofilm gene expression. For instance, removing eDNA disrupts the environmental conditions that sustain the expression of *sarA*, *sigB*, and *icaADBC*, thereby reducing matrix production. *In vivo* studies using murine models of device-related infections have demonstrated that DNase I reduces *S. aureus* biofilm burden and enhances the efficacy of antimicrobial therapy (Table 5; Bowden et al., 2024). Furthermore, the synergistic use of DNase I with immunomodulators or antibiotic-loaded nanoparticles (NPs) enhances biofilm eradication, offering a translational avenue for treating chronic wounds and implant infections. These findings underscore the therapeutic potential of DNase I as a non-antibiotic adjunct capable of dismantling the biofilm architecture and re-sensitizing bacteria to host and pharmacological defenses (Deng et al., 2024).

**Table 5:** Emerging therapeutic strategies targeting *S. aureus* biofilms: molecular targets, mechanisms, translational evidence, limitations, and future prospects.

Strategy	Primary molecular target	Mechanism of action	Current level of evidence	Advantages	Major limitations	Clinical status	Future prospects	References
DNase I	eDNA	Hydrolyzes eDNA, destabilizing the biofilm matrix and improving antibiotic penetration	*In vitro*; multiple animal models	Effective during early biofilm formation; synergistic with antibiotics; low likelihood of resistance	Limited efficacy in protein- or PNAG-rich mature biofilms; short half-life; requires local delivery	Preclinical	Develop sustained-release formulations and combination therapies	Mann et al. (2009); Tetz et al. (2009)
Dispersin B	PNAG/PIA matrix; applicable primarily to PNAG-dependent biofilms	Cleaves β-1,6-linked poly-N-acetylglucosamine, disrupting matrix integrity	*In vitro*; animal studies	Selective matrix degradation in PNAG-dependent biofilms; can enhance antibiotic activity	Limited efficacy against PNAG-independent protein/eDNA-dominant biofilms; efficacy depends on PNAG abundance and accessibility; enzyme stability and immunogenicity require evaluation	Preclinical	Matrix-guided therapy and combination with DNase/protein-targeting approaches for mixed or PNAG-independent biofilms	Kaplan et al. (2025)
Protein-matrix disrupting enzymes	SasG/Bap/FnBPs and other matrix-associated proteins	Proteolytic cleavage of proteinaceous matrix components, weakening intercellular cohesion and promoting detachment	Mainly *in vitro*; limited *in vivo* evidence	Targets PNAG-independent protein-rich biofilms; complementary to PNAG-directed and antibiotic therapies	Potential host-tissue toxicity, nonspecific proteolysis, limited stability and delivery	Experimental/preclinical	Development of biofilm-selective proteases and combination therapies guided by matrix composition	Crosby et al. (2024)
Proteolytic enzymes (Proteinase K, Trypsin, Serratiopeptidase)	Proteinaceous matrix components (SasG, Bap where present, FnBPs and other surface-associated proteins)	Degrade proteinaceous biofilm scaffold and promote bacterial detachment	Mainly *in vitro*; limited animal data	Effective against protein-rich biofilms; potentiates antibiotics	Host tissue toxicity; nonspecific proteolysis; short *in vivo* persistence; limited penetration into mature biofilms; potential immunogenicity	Experimental	Develop biofilm-selective proteases with improved specificity	Boles and Horswill (2008); Kumar Shukla and Rao (2013)
Quorum-sensing inhibitors (Savirin, Solonamide B, RNAIII inhibitors)	*agr*-quorum-sensing system (AgrA/AgrC/RNAIII)	Suppress virulence gene expression, biofilm dispersal, and toxin production	*In vitro*; limited animal studies	Reduce virulence without directly inhibiting bacterial growth; lower selective pressure for resistance	Strain-dependent *agr* genotype and functional status; *agr* I–IV sequence variation; background-dependent signaling; potential reduced efficacy in *agr*-dysfunctional isolates	Preclinical	*agr* genotyping and functional phenotyping to guide therapy; combination approaches tailored to the regulatory state of the infecting strain	Nielsen et al. (2014); Sully et al. (2014)
Antimicrobial peptides (AMPs)	Bacterial membrane and biofilm matrix	Disrupt membrane integrity, penetrate biofilm matrix, and modulate immune responses	*In vitro*; animal studies; few early clinical investigations for wound applications	Broad-spectrum activity; rapid bactericidal effect; low propensity for resistance	Proteolytic degradation; potential cytotoxicity; high manufacturing cost	Mostly preclinical	Improve peptide stability through nano-formulation and peptide engineering	Dean et al. (2011); de Breij et al. (2018)
Bacteriophage therapy	Viable bacterial cells within biofilms	Phage-mediated bacterial lysis with self-amplification inside biofilms; often synergistic with antibiotics	Extensive *in vitro* studies; multiple animal models; compassionate-use reports and early clinical studies	High specificity; effective against multidrug-resistant strains; biofilm penetration; self-replicating	Narrow host range; emergence of phage resistance; regulatory and manufacturing challenges	Early clinical evaluation	Standardized phage cocktails, randomized clinical trials, and optimized phage–antibiotic combinations	O’Flaherty et al. (2005b); Cerca et al. (2007); Mobarezi et al. (2025)
Phage-derived lysins (e.g., LysK)	Peptidoglycan cell wall	Rapid enzymatic cleavage of peptidoglycan causing bacterial lysis	*In vitro*; animal models	Rapid bactericidal activity; active against dormant cells; synergy with antibiotics	Limited Gram-negative activity; possible immunogenicity; repeated dosing required	Preclinical	Protein engineering to improve stability and serum half-life	O’Flaherty et al. (2005a); Gu et al. (2011)
Nanoparticle-based delivery systems	Biofilm matrix and bacterial cells	Enhance targeted drug delivery, sustained release, and penetration through EPS	*In vitro*; animal studies	Improved drug bioavailability; controlled release; reduced systemic toxicity	Unpredictable biodistribution and biofilm penetration; long-term biocompatibility; payload stability/loading; scalable GMP manufacturing; and regulatory approval	Preclinical	Stimuli-responsive and multifunctional NPs with clinical validation	Hussain et al. (2018); Yang et al. (2018)
Nitric oxide (NO)-releasing systems	Intracellular signaling pathways regulating biofilm dispersal	Induce biofilm dispersal and increase susceptibility to antibiotics	Primarily *in vitro*; limited animal studies	Broad activity; enhances antibiotic efficacy; promotes wound healing	Difficult control of NO release; potential host toxicity at high concentrations	Experimental	Controlled-release biomaterials and combination therapy	Carpenter et al. (2012)
CRISPR-Cas-based antimicrobial systems	Essential genes, resistance determinants, or virulence genes	Sequence-specific cleavage of target genes causing bacterial killing or virulence attenuation	Proof-of-concept *in vitro*; limited animal studies	High specificity; programmable targeting; potential to eliminate resistance genes	Delivery remains a major challenge; off-target concerns; regulatory complexity	Experimental	Phage-mediated CRISPR delivery and precision antimicrobial therapy	Bikard et al. (2014)

Clinical trial consideration. Across these therapeutic classes, future clinical studies should prospectively define infection-specific clinical, microbiological, and recurrence endpoints and, where feasible, incorporate validated pharmacodynamic measures of biofilm disruption. Mechanistic reductions in biofilm biomass or matrix components should be interpreted as supportive pharmacodynamic evidence rather than assumed surrogates of clinical cure.

However, issues such as exposure time, enzymatic stability, tissue distribution, and local retention are important considerations for the clinical translation of DNase I and other matrix-degrading enzymes (Tian et al., 2023). They are effective when they have good access to the biofilm matrix, but may have insufficient activity after systemic delivery when the biofilm is quickly cleared or degraded. Stabilization of enzymes and directed delivery to the site of infection may therefore be necessary to ensure that sufficient levels of the enzyme remain in the area of infection and in the bloodstream (Li et al., 2023). These factors are especially critical in deep-seated biofilm infections, in which topical or device-localized delivery options may be available in select cases, and systemic or image-guided options may be required for anatomically inaccessible infections.

The emerging antibiofilm drugs against *S. aureus* include the molecular targets and mechanisms summarized in Figure 4. These strategies include targeting components of the ECM, quorum-sensing pathways, the bacterial cell envelope, or key bacterial intracellular genes, all of which facilitate biofilm disruption, increase antibiotic efficacy, reduce bacterial persistence, and improve therapeutic outcomes in chronic biofilm-associated infections.

**Figure 4:**
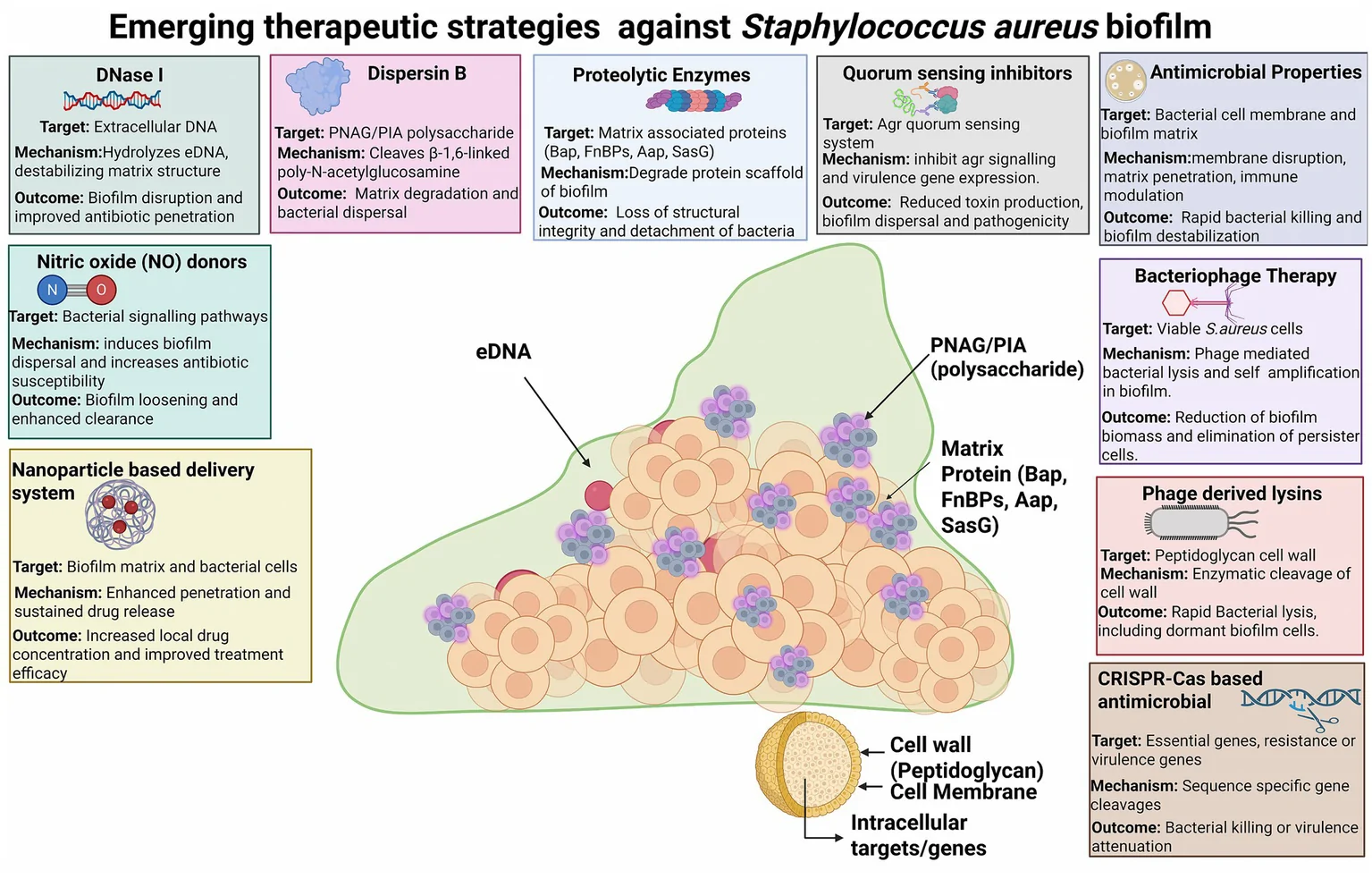
Molecular targets and mechanisms of novel therapeutics targeting *S. aureus* biofilms. The figure shows the main antibiofilm approaches to the structural and regulatory aspects of *S. aureus* biofilms. These include matrix-targeting enzymes directed against eDNA (DNase I), PNAG/PIA (Dispersin B), and proteinaceous matrix components; quorum-sensing inhibitors; antimicrobial peptides; nitric oxide donors; nanoparticle-based drug delivery; bacteriophage therapy; bacteriophage-derived lysins; and CRISPR–Cas antimicrobials. Together, these strategies can alter biofilm structure, increase antimicrobial penetration, kill persister cells, and reduce bacterial virulence.

#### 5.2.2 Dispersin B: degrading PIA

Dispersin B is a glycosyl hydrolase enzyme isolated from an *Aggregatibacter actinomycetemcomitans* and is specific to the hydrolysis of PNAG, the major exopolysaccharide secreted by an *icaADBC* operon in *S. aureus* biofilms (Kaplan et al., 2024). Acting on the glycosidic bond of PNAG, dispersin B alters the matrix structure by breaking it down and releasing bacteria within the biofilm (Breslawec et al., 2021). Microbiologically, this enzymatic effect disrupts the defensive network that restricts antibiotic access and phagocytic cell deposition, thereby exposing biofilm-inhabiting *S. aureus* to immune and antimicrobial activity. It has been demonstrated that Dispersin B reduces biomass and enhances the activity of drugs such as daptomycin and linezolid, especially against multidrug-resistant strains (Ramakrishnan et al., 2022). At the molecular level, Dispersin B activity indirectly interferes with PNAG, thereby altering transcription of biofilm regulatory genes. Among the global regulators that control biofilm persistence and stress response are *sarA* and *sigB,* whose dysregulated expression may drive biofilm-related bacteria toward an aplanktonic adulthood.

Dispersin B hydrolyzes PNAG/PIA, thereby specifically attacking PNAG-dependent biofilm matrices. Dispersin B can degrade PNAG, reduce intercellular cohesion, reduce bacterial biofilm biomass, and increase bacterial exposure to antimicrobials (Dabul et al., 2025). Experimental studies have demonstrated that PNAG-targeting approaches can be successfully combined with conventional therapy, resulting in even better antibiotic activity (Fanaei Pirlar et al., 2020; Temme et al., 2024). However, its activity depends on the presence and availability of PNAG and should therefore not be viewed as a general approach to disrupting biofilms. The susceptibility of PNAG-independent biofilms may be significantly lower to Dispersin B because protein- or eDNA-dominant biofilm matrices that rely on SasG, Bap, protein A, and FnbA/B may not be susceptible to the enzyme’s substrate (Garmendia-Antoñana et al., 2026). Therefore, choosing or designing antibiofilm therapies should take into account the composition of the matrix, such as the PNAG-positive/PNAG-dependent phenotype, which is best targeted by dispersin B, or protein-based, DNase-based, or combined matrix-targeting therapies, and are more effective for protein or eDNA-dominant phenotypes (Dabul et al., 2025). This matrix-dependent therapeutic principle underscores the need for mechanism-based antibiofilm drug therapy, rather than targeting a single, common extracellular scaffold across all *S. aureus* biofilms.

Translationally, Dispersin B represents the general pharmacokinetic challenges of antibiofilm therapy with enzymes. It must be physically present in the PNAG within the biofilm matrix and have sufficient dwell time at the infection site. However, its efficacy after systemic administration may be limited by rapid clearance, proteolytic degradation, and poor penetration into heterogeneous or deeply embedded areas within the biofilm (Kaplan et al., 2025). Furthermore, the activity of Dispersin B is matrix-dependent as it is highly specific for PNAG and can be lowered in PNAG-independent protein- or eDNA-dominated biofilms (Garmendia-Antoñana et al., 2026). Therefore, future clinical development may focus on stabilized or targeted delivery and, where anatomically suitable, local delivery, ideally in combination with antibiotics and, if needed, other matrix-disrupting agents.

Further, the therapeutic potential of Dispersin B is likely to be strain- and matrix-dependent, because PNAG is not the predominant structure in all *S. aureus* biofilms (Kratofil et al., 2026). However, strains producing low amounts of PNAG or matrix dominated by proteinaceous/eDNA may show a limited response to PNAG-mediated matrix disruption (Hao and Wang, 2025). Importantly, the presence of the *icaADBC* gene does not necessarily mean the strain is “genetically and environmentally competent” for PNAG-dependent growth, as expression depends on the strain’s genetic and environmental background (Syed et al., 2024). Therefore, knowledge of the matrix composition or the presence of PNAG may be required to determine which infections are most likely to respond to Dispersin B-based therapy.

#### 5.2.3 Targeting the agr system and quorum-sensing

*S. aureus agr* quorum-sensing system regulates the sessile biofilm to planktonic phase transition, virulence factor production, and population-level behavior. The *agr* locus is a two-component (agrC-agrA) and AIP quorum-sensing system (Tan et al., 2022). Together, these components facilitate gene regulation in response to cell density. During planktonic cells and acute infections, the active *agr* signaling stimulates expression of extracellular proteases, toxins (e.g., alpha-hemolysin), and PSMs. It inhibits surface adhesins and biofilm matrix products. Downregulation or mutation of *agr*, which is common in chronic and device-related infections, on the contrary, contributes to increased biofilm production by augmenting transcription of *fnbA*, *spa*, *icaADBC,* and other matrix-related genes (Cheung et al., 2014). Pharmacological *agr* targeting is possible by inhibiting AIP production, interfering with the AIP-agrC interaction, or disrupting downstream signaling through agrA. RNAIII-inhibiting peptides, such as savirin (*S. aureus* virulence inhibitor), and solonamide B analogs are examples of molecules shown to reduce *agr*-mediated gene expression (Nielsen et al., 2014). The agents decrease toxin secretion, biofilm dispersal, and inflammation, but there is no direct selective pressure on bacterial growth, thereby reducing the development of resistance (Vasquez and Blackwell, 2019). Microbiologically, *agr*-inhibition promotes biofilm attachment and stability and reduces immunopathology by restricting cytotoxin production; thus, *agr*-inhibitors are especially appealing for treating infections that prompt rapid inflammation and tissue damage, e.g., endocarditis or pneumonia. In addition, *agr* inhibitors and antibiotics could be used together to prevent early biofilm resuspension and reduce the loss of virulent planktonic cells during therapy, a factor associated with metastatic infection (Jordan et al., 2022). On the molecular level, *agr* repression alters the entire *S. aureus* regulatory network and usually directly increases *sarA* and *sigB* activity, which regulate biofilms and the stress response in the bacteria. Though this transition favors biofilm persistence, it also creates vulnerabilities, including a heightened reliance on ECM components and an impaired ability to evade the immune system via toxin-induced lysis (Paharik and Horswill, 2016). These downstream effects provide therapeutic windows in which synergy with *agr* inhibitors can be achieved through matrix-disrupting proteins (e.g., Dispersin B) or immune-enhancing molecules. Inhibitors of quorum-sensing therefore have two advantages: the first is that they reduce virulence, and the second is that they help reconfigure the biofilm’s physiology in a manner that is therapy-exploitable without exerting significant selective pressure, as would be the case with conventional antibiotics (Table 5; Gray et al., 2013).

Results of *agr*-directed therapy may probably be strain-specific. Four major allelic groups of the agr locus are known, and there is considerable sequence variation in agrB, agrD, and agrC, which accounts for the specificity of the AIP and the quorum-sensing behavior of each group (Bär et al., 2026). Furthermore, naturally occurring agr dysfunction may affect the balance among adhesin expression, matrix production, proteolysis, and toxin-mediated dispersal (Ding et al., 2023). The effects of replacing various agr alleles have been studied experimentally, and the results suggest that the phenotypic outcome is strongly dependent on the background strain. This means that the *agr* genotype is not a good predictor of therapeutic outcome (Pivard et al., 2026). Thus, *agr* typing, evaluation of *agr* activity, and complete regulatory information for the infecting strain should be included in the future clinical development of *agr*-targeting agents.

#### 5.2.4 Nanoparticle-mediated delivery and phage therapy

One of the biggest problems with conventional and macromolecular antibiofilm is that the pharmacokinetics of the drugs in the bloodstream does not match the pharmacology in the biofilm. Therapeutic molecules are required to penetrate the ECM, penetrate bacterial populations in deeper tissues, and be maintained at therapeutic levels, even in the presence of diffusion barriers, enzymatic degradation, and clearance. This is especially important in “persister” regions of mature biofilms with low metabolic activity that further diminishes the effectiveness of antibiotics (Bin et al., 2025). Nanoparticle-based delivery systems can thus be used to deliver drugs that may provide increased local drug concentration, protect labile payloads, extend the timescale for drug action, and facilitate penetration into biofilm-associated niches (Cui et al., 2024). Several NPs [e.g., liposomes, polymeric NPs (e.g., PLGA), metallic NPs (e.g., silver and gold), and lipid-based nanocarriers] can be personalized to deliver antimicrobial substances in a precise, sustained, and stimuli-responsive manner (Xia et al., 2025). Targeted delivery systems may also take into consideration the inter-strain variation in the abundance, accessibility, and sequence conservation of their molecular targets. As a result, ligands targeting PNAG or FnBPs, protein A, or other surface-associated determinants may exhibit differential binding between clinical isolates, while sequence-specific ligands, like CRISPR-Cas antimicrobials, can be especially sensitive to nucleotide variation in the selected target sequence as well as the surrounding regions (Coll et al., 2026). Therefore, the design and microbiological characterization of strain-specific or precision antibiofilm therapies should ideally take into account the target-specific, expression, and accessibility.

Research findings demonstrate that NPs with vancomycin, rifampicin, or daptomycin exhibit increased biofilm penetration and bactericidal effects; hence, they disrupt the regulatory pathways of biofilm, such as *sarA* and *sigB*, which play an essential role in biofilm stability. Furthermore, the formation of ROS by metallic NP breaks the biofilm matrix and the bacterial membrane (Kim et al., 2023).

Simultaneously, as an alternative or supplement to antibiotics, bacteriophage (phage) therapy can be used (a form of therapy that uses viruses that specifically infect and lyse bacteria). Lytic phages belonging to the *S. aureus* species can penetrate biofilms and kill bacteria regardless of their metabolic activity, thereby addressing antibiotic tolerance (Pires et al., 2017). Some of these phages and phage products are known biofilm-degrading enzymes, e.g., depolymerases or endolysins, that degrade EPS and cell walls, creating deeper access into the biofilm and increasing the effectiveness of combined phage and antibiotic co-treatment (Table 5). Phage infection can circumvent several resistance mechanisms at the molecular level, since, unlike antimicrobials such as penicillin, phage directly target bacterial nucleic acids or peptidoglycan (Fenton et al., 2013). There has also been the development of engineered phages loaded with R-Cas systems or antibiofilm protein genes, which can broaden therapeutic applications (Ali Agha et al., 2025). Experiments in living organisms, using phage cocktails or phage-loaded NPs, have demonstrated promising prospects for personalized, precision-based antimicrobial therapy, with MRSA biofilms eliminated from implants, wounds, and respiratory tissues (Malik et al., 2017).

Host immunogenicity is another consideration while using recombinant phage-derived lysins. The systemic or repeated use of lysins may lead to the formation of anti-drug antibodies, which can enhance the clearance of these agents or lower the enzymatic activity. Antibody responses have been observed in human and preclinical studies, but the ability to neutralize antibacterial activity varies widely among lysins (Bitoun et al., 2023). For chronic infections that require repeated dosing, this poses a potential exposure–response problem, as antibody titers may increase without a corresponding increase in the active drug concentration. Therefore, next-generation lysins need to be evaluated for immunogenicity during development, including anti-drug antibody formation, neutralizing activity, repeat-dose pharmacokinetics, and inflammatory or hypersensitivity responses (Fowler et al., 2024). This liability can be minimized by rational epitope engineering and strategic protein modifications or delivery platforms while maintaining catalytic activity.

## 6 Conclusion

The formation of *S. aureus* biofilm is a highly regulated and dynamic process involving temporally regulated interactions among adhesins, ECM components, global transcription regulators, TCSs, and environmental *stimuli*. The regulatory networks between *icaADBC*, *sarA*, *agr*, *sigB*, *saeRS*, and *arlRS* work together to control the initiation, maturation, persistence, and dispersal of biofilm, as well as metabolic adaptation, immune evasion, and antibiotic tolerance. A combination of these molecular mechanisms, transcriptomic reprogramming, and clinically relevant biofilm phenotypes yields a global view of how *S. aureus* develops chronic, recurrent, and device-associated infections across various host environments.

While traditional antibiotics remain the mainstay of treatment, their efficacy is significantly diminished in mature biofilms, underscoring the need for mechanism-based therapeutic interventions. New strategies to overcome biofilm-associated persistence include tools to disrupt the ECM (e.g., enzyme-based disruption), antimicrobial peptides, bacteriophages, phage-derived lysins, nanoparticle-assisted drug delivery, NO donors, and CRISPR–Cas systems.

Future studies should prioritize clinically validated combination therapies together with infection-site-specific delivery platforms, pharmacokinetic/pharmacodynamic optimization, and systems-level approaches that account for biofilm heterogeneity, tissue penetration, and regulatory networks across infection stages. Demonstrating sustained therapeutic exposure within mature biofilms while maintaining acceptable systemic and local safety may be essential for translating promising preclinical antibiofilm strategies into routine clinical practice.

For protein-based antibiofilm therapeutics, future development should additionally prioritize repeat-dose immunogenicity, anti-drug antibody neutralization, and exposure–response relationships to ensure that sustained therapeutic activity is maintained during prolonged treatment.

## Glossary

Aap: Accumulation-associated protein
AMPs: Antimicrobial peptides
AIP: Autoinducing peptide
*agr*: Accessory gene regulator
Atl: Autolysin
ArlRS: Autolysin-related locus regulatory system
Aur: Aureolysin
Bap: Biofilm-associated protein
ClfA/B: Clumping factor A/B
Cna: Collagen-binding adhesin
eDNA: Extracellular DNA
ECM: Extracellular matrix
EPS: Extracellular polymeric substance
FnbA/B: Fibronectin-binding protein A/B
*icaADBC*: Intercellular adhesin operon
*icaR*: Repressor of icaADBC
KatA: Catalase
MRSA: Methicillin-resistant *Staphylococcus aureus*
MSCRAMMs: Microbial surface components recognizing adhesive matrix molecules
NPs: Nanoparticles
Nuc: Nuclease
PIA: Polysaccharide intercellular adhesin
PLGA: Poly(lactic-co-glycolic acid)
PNAG: Poly-N-acetylglucosamine
PSMs: Phenol-soluble modulins
ROS: Reactive oxygen species
rsp: Regulator of SarA pathway
sarA: Staphylococcal accessory regulator A
SaeRS: *S. aureus* exoprotein expression regulator
SCV: Small colony variant
sigB: Sigma factor B
SodA: Superoxide dismutase
spa: Staphylococcal protein A
sspA: Serine protease
TCSs: Two-component systems
